# A Realist-Informed Review of Digital Empowerment Strategies for Adolescents to Improve Their Sexual and Reproductive Health and Well-being

**DOI:** 10.1007/s11524-022-00678-8

**Published:** 2022-09-07

**Authors:** Kunshan Goh, Sana Contractor, Sara Van Belle

**Affiliations:** 1 Amsterdam University Medical Centers, Amsterdam, Netherlands; 2 Center for Health and Social Justice, New Delhi, India; 3 Institute of Tropical Medicine, Antwerp, Belgium

**Keywords:** Realist synthesis, Realist review, Empowerment, Digital health, Adolescents, Sexual and reproductive health and rights

## Abstract

The use of digital technologies for health has been rapidly gaining ground in the last decade, including as a strategy to empower adolescents living in urban resource-constrained settings. Nevertheless, unclarity and incoherence remain regarding which programme strategies generate which outcomes, as well as regarding the importance of context. We set out to answer the question “How do digital empowerment strategies work to improve adolescent health and well-being?”. We conducted a realist-informed review, the first on this subject matter to our knowledge. The realist methodology is geared towards the understanding of socially complex interventions, such as digital empowerment. We synthesized the data into a programme theory uncovering social mechanisms and context conditions underlying specific programme strategies. We found that digital technologies enlarge the space for adolescents to access information to health services on their own terms and provide anonymity, which leads to a sense of safety if access is not curbed by gatekeepers. If adolescents have access to information adapted to their needs, they will be able to make informed decisions, and this will contribute to improved health outcomes because their better understanding enlarges their sense of individual agency. We identified two main gaps in the literature on digital interventions for adolescents. Both are related to an under-theoretisation of the concepts the programmes rely on in implementation: (1) the urban environment the programme has to operate in and its meaning for the adolescents; (2) the socio-developmental stage of the adolescents the programmes work in.

**Supplementary Information:**

The online version contains supplementary material available at 10.1007/s11524-022-00678-8.

## Introduction

The use of digital technologies for health has been rapidly gaining ground in the last decade, including as a strategy to empower adolescents living in urban resource-constrained settings. Adolescents are at a crucial juncture in their life and often lack the psychological and physical safety both in the urban public and private space [1], which is detrimental to their sexual and reproductive health and overall health and well-being [2]. They also often lack the skills to negotiate relationships, claim accountability, and advocate for their needs in a complex urban environment [3, 4]. Strengthening their voice and agency through the use of digital technologies could potentially mobilise individual and collective action, thereby possibly improving sexual and reproductive and overall health and well-being [5].

Nevertheless, unclarity and incoherence remain regarding which programme strategies generate which outcomes, and the role of context conditions is equally under-researched. In the field of health promotion, and of adolescent sexual and reproductive health and rights specifically, many interventions to support adolescent health now include a digital or social media component [6, 7]. Such digital empowerment for health interventions includes a broad range of modalities, implementation strategies, context conditions, and outcomes. However, many interventions have been exclusively documented through grey literature, mostly by way of programme evaluations of international NGOs. As within other programmes, there is an urgent need to better understand how these programmes work, and under which context conditions they produce beneficial outcomes to adolescent health.

Programmes using digital empowerment addressing adolescents living in urban resource-constrained settings in lower and middle-income countries (LMIC) fit the bill of a complex intervention, where social change is envisaged through an intervention embedded in a complex web of social and cultural structures and norms [8]. The UK Medical Research Guidance recently updated guidance on the evaluation of complex interventions [9]. Research on complex interventions intends to encompass a broader range of goals beyond impact, such as the objective of theory-building regarding how and what works, the resources required, and analysis of the context conditions in which the intervention worked [8].

To address the gap in knowledge, we conducted a realist-informed review. Our initial review question was “how do digital empowerment strategies work to improve adolescent health and well-being?” In this review, we included the interventions which use digital technology, be it software (apps), hardware (mobile phone), or both, as strategies to achieve adolescent health outcomes.

We consider this literature review a realist-*informed* review, as this is an emergent, rapidly evolving, and scattered field where evaluations of interventions have mainly been published in grey literature and theorization is still sparse. Our ambition was to explore a broad scope of literature to identify current trends and identify knowledge gaps as well as to formulate an initial programme theory, without aiming at being exhaustive. The review is part of a broader research project “Msichana -Chakti!” on local accountability ecosystems to strengthen sexual and reproductive health and rights of adolescent girls living in informal settlements in India, Uganda, and Benin.

## Methods

Our rationale for undertaking a realist-informed review was to better understand how digital empowerment strategies, embedded in complex programmes, could contribute to improved adolescents’ health and well-being. Realist review is a form of evidence synthesis that hails from the theory-driven school of evaluation and which goes beyond examining the outcomes in terms of the effectiveness of interventions to understand why and how a complex intervention in a given context produces certain outcomes [10, 11]. It has steadily gained ground in health systems and policy research and is geared towards a better understanding of socially complex interventions beyond “simply” assessing effectiveness [12]. The realist method gives due attention to the variety of outcomes that can be generated when a complex intervention works in different social environments. It seeks to identify how actors’ practices are emerging in specific context conditions and aims to explain why this happens, through searching for causal powers which are embedded in mechanisms [13]. In effect, mechanisms are what trigger the actors’ actions in a given context. The result of a realist review is the synthesis of evidence in the form of an initial programme theory (IPT), which in a subsequent phase can be empirically tested [6, 14].

We sought to formulate plausible explanations in the form of patterns that consist of sets of social mechanisms and context conditions. Realists use a heuristic tool called the Context-Mechanism-Outcome configuration or variants such as the Intervention-Context-Actors-Mechanism-Outcome configuration (ICAMO) [6, 15]. ICAMOs represent the synthesis of results from specific studies and through theoretical abstraction they can be reformulated at the level of the “programme theory”. This in turn can provide valuable information for future programme design [6]. Realist researchers aim to contribute to a cycle of theory-building that transcends separate programmes, leading to ever more knowledge in a certain domain regarding how programmes trigger sets of social mechanisms in different sets of context conditions to lead to specific outcomes [13].

We followed the six-step framework recently published by Hunter et al. [16] and the realist review methodological guidance provided by the RAMESES project [6]. Originally applied to a realist review of socially complex interventions in the domain of sports psychology, Hunter et al. provide a clear overview of six stages of the review process. Their framework takes the RAMESES guidance into account and contributes to further clarifying methodological guidance and enhancing transparency of the application of the method [16].

The six steps include the following: (1) the identification of the research question; (2) the search for evidence; (3) the selection of documents (quality appraisal); (4) data extraction; (5) data synthesis; and (6) dissemination of findings. These key steps are summarized in Table 1. We will discuss the results for each of the steps in the next section. The review process, excluding writing up, included the months of February until June 2021.

**Table 1:** Key steps of the realist review process

Step 1: Identifying the research question	Identified research and knowledge gap present in larger project, review question developed by review team; informal literature search, mining concepts, and theories; set of hypothetical statements in the form of a “rough” or starter IPT
Step 2: Searching for evidence	Refinement search terms and conducted systematic literature search initially in (1) databases and (2) grey literature; scope was adapted and expanded to include non-urban contexts (iterative process)
Step 3: Document selection (quality appraisal)	Final selection of 26 papers (Fig. 2)
Step 4: Data extraction	Iterative process of data extraction; emergent themes discussed by review team, data coded, final data extraction sheet developed
Step 5**:** Data synthesis	Coded data extracts within the ICAMO framework were iteratively refined with inputs from review team, leading to a synthesized IPT
Step 6: Reporting and dissemination of findings	Findings will be disseminated within context of broader research project *MSICHANA/CHAKTI!*

## Results

- Step 1: Identifying the Review Question

The first step of the framework of Hunter et al. does not only refer to the identification of the research question, but also the clarification of underlying assumptions, the mining for concepts, and substantive theories culminating in a set of hypothetical statements or “rough” IPT [16]. This corresponds with the general guidance that initial programme theories can be formulated in the basis of eliciting the assumptions of stakeholders (also called folk theories) and research, and existing evidence and theories [13]. In addition, we also formulated “rival” programme theory clauses, as it is good practice to search for rival explanations of effects [17].

Adolescents living in informal settlements or poor neighbourhoods lacking in public services experience difficult conditions at a time they are transitioning into adulthood. This puts them at risk of sexual and reproductive ill health, psychological vulnerability, and sexual violence, in or outside their homes [18, 19]. Many girls at this age are learning to negotiate relationships, while at the same time facing unwanted pregnancies and forced early marriages, or are being compelled to engage in sex work for economic motives [20]. Often, these adolescent girls do not have access to basic health and social services catering to their specific developmental needs [21].

We set out to better understand the role of varying context conditions in digital empowerment interventions to support the health and well-being for adolescents living in poor, resource-constrained urban environments. The rationale is that increasingly, digital communication tools, such as digital apps, are seen as a promising instrument to support adolescents’ voice and agency, thereby transforming their accountability relationships, with the potential outcome of improved health and well-being [5].

We wanted to explore two interrelated paths with the review. First, we reviewed the expectations of how such a programme is expected to work against actual practice. Most of the time, high expectations are attached to introducing innovative elements in NGO programmes such as digital media. One expects technology sometimes to work as a magical solution for a number of issues, or to be without risk [22, 23]. Second, health interventions using digital communication technology are often transferred from one setting to another without much adaptation, as programme developers sometimes build their programmes on the idea that adolescents across settings are living in similar digital worlds, and that these do not much interact with their needs in the real world [20].

### Clarifying our Underlying Assumptions

The review team consisted of one junior researcher from Singapore, an occupational therapist with a MSc degree in global health now working on an adolescent-led co-creation project for adolescent health in a poor neighbourhood of Amsterdam. The second author is a public health researcher and practitioner who has worked for 15 years on collective action for health with organisations in urban and rural India, furthering sexual and reproductive health and rights with a focus on citizenship. She is also a coordinator of a global network on accountability in sexual and reproductive health. The third member of the review team is a European senior researcher-social scientist who has worked in Africa for NGOs in sexual and reproductive health and rights in programme management, policy, and advocacy for 10 years, and then pursued academic research in sexual and reproductive health and rights (SRHR). We knew from our professional experience that many current programmes, even when working with and for adolescents in informal settlements, are using digital tools to support adolescents’ health and well-being, which was confirmed when reviewing the more recent literature.

One of our assumptions is that in actual practice, to be able to ‘work’, digital interventions require continuous adaptation to the setting, the group of adolescents and the resources available. A second assumption points to the likelihood of unintended effects, since digital media use is very much part of adolescents’ lives in one way or another in LMIC as well as in HIC and the implementation of digital strategies will be very much intertwined with adolescents’ digital media use.

### The Research Questions

We formulated the following review questions: (1) how are digital interventions used to support adolescents’ sexual and reproductive health and well-being impacted by differing context conditions?; (2) who were the actors playing the most important roles impacting on the success of a digital intervention? and (3) if context plays an important role, how did the programmes reported on taking this into account, and did the programme components change as a result?

We limited the study group to adolescents, which we defined as within the age group 10 to 19 years [24]. For the purpose of the review, we define empowerment as “a personal journey during which an adolescent through increased assets and critical awareness develops a clear and evolving understanding of themselves, their rights and opportunities in the world around them, and through increased agency, and voice and participation, have the power to make personal and public choices for the improvement of their lives and their world.” [25].

Given that empowerment is a term with a broad range of meanings, we included interventions that have empowerment as a programme strategy (digital empowerment), as well as those that consider empowerment of adolescents as an outcome [5]. We included interventions on adolescents sexual and reproductive health and mental health as well as during this phase of adolescent transition, attention to adolescents’ mental health is key [6, 26].

We defined digital technology as electronic systems, devices, applications, and tools such as social media, online games, multi-and interactive media, mobile phones, tablets, laptop, and desktop computers. It has been found that digital technology not only supports advances in health, but also has human rights implications in relation to individual privacy, security, and agency [27].

### Concept Mining

Another element within step 1 of Hunter et al. concerns the mining and clarification of concepts used in the review. As this is an emergent field, we found indeed a lack of conceptual clarity and coherence of evidence during our initial scoping of the literature.

We conducted an informal literature search mining for concepts. Conceptual terms used in this review correspond to our perspective on donor-funded programmes in the field of adolescent and young adult sexual and reproductive health and rights, with their current focus to improve accountability towards adolescents through participatory approaches, and the use of digital technology to boost adolescents’ individual and collective empowerment [5, 28]. The selection of search terms was influenced by the conceptual overview (Annex 1).

### Search for Substantive Theories

We searched for social science theories regarding digital communication and adolescents and accountability in LMIC, including Deci and Ryans’ self-determination theory and adolescent development from social psychology [29], theories regarding accountability for adolescents [30], and frameworks on youth-friendly adolescent sexual and reproductive health services [31].

It is not an exaggeration to state that the field of adolescent empowerment suffers from a lack of adolescent engagement and under-theoretization. Programme funders or developers are often not explicitly applying evidence or lessons learnt, nor are they describing the underlying mechanisms or theories. What Vincent et al. reported in their review on community engagement in malaria research holds here as well; adolescent health programmes suffer from “poor abstraction”, where terms such as trust, confidence, urban, and the like are used colloquially without proper definition or specification according to context conditions [14].

Finally, there is often no time nor resources to adapt programmes to the needs of the urban adolescents in that specific setting as stated by Tuhebwe et al. in relation to SRH programmes and adolescent needs in Kisenyi, an urban poor setting in Kampala, Uganda [21].

### “Rough” or Starter IPT

The initial starter or “rough IPT” was translated into hypothetical “if–then-because statements”, resulting in the following [4]:

- “If digital empowerment tools and resources are included in programmes as a transformative accountability strategy,
- Then this will lead to strengthened voice and agency, which will lead to the outcome of improved SRHR outcomes and well-being for adolescents in resource-constrained settings,
- Because adolescents’ empowerment and transition to adulthood plays out partially in and through the digital world, or the world accessible through the use of digital technologies.”

Three sets of potential rival programme clauses were discussed by the review team, in accordance with Pawson et al. [17]:

- The first set of clauses pertains to the causal linkage between the use of digital technology and empowerment. One rival clause here is that digital strategies on their own do not lead to strengthened voice and agency, but need to be accompanied by other non-digital empowerment strategies. Another related potential rival clause is that digital empowerment might lead to strengthened individual voice and agency, but ignores collective voice and collective agency.
- The second set relates to the causal linkages between strengthened voice on the one hand, and improved health and well-being outcomes. For example, it might be possible that there is no effect health and well-being outcomes.
- The final set pertains to the context conditions. In certain settings, such as remote rural areas or informal settlements in LMIC, intermittent internet access may pose a limiting effect on the effectiveness of digital strategies.

**Fig. 1:**
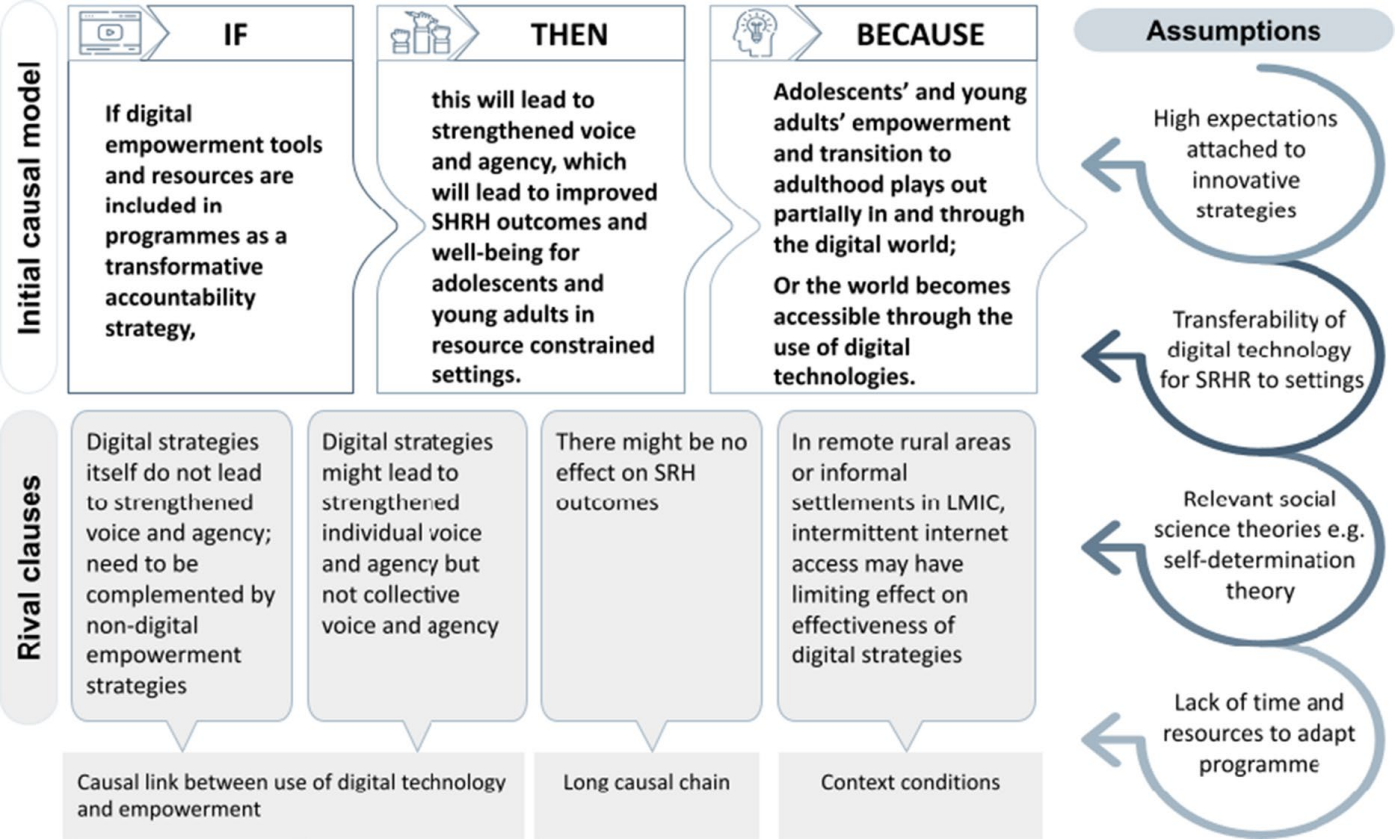
The “rough” or starter IPT

A visual representation of the “rough” or starter initial programme theory, rival clauses, and underlying assumptions which position this realist review is presented in Fig. 1.

- Step 2: Searching for Evidence

The search for evidence was an iterative process, common to realist review. In step 1, we conducted an initial search to obtain a better understanding on how to best demarcate the review topic and question. In Step 2, we designed and piloted our search strategy. The pilot of the search strategy learned us there is a dearth of academic peer reviewed literature on this emerging topic, and as a result, we decided to expand our search to high-income countries and to include rural zones.

We defined the search terms and formulated combinations, as presented in Table 2.

**Table 2:** Search term combinations

‘digital technolog*’ OR ‘cell phone use’ OR ‘web-based intervention*’ OR ‘social media’ OR ‘mobile phone’ OR ‘ICT’ OR Internet OR digital media OR ‘DCT’ OR ‘online’
AND
'adolescent*' OR 'youth*' OR 'young adult*' OR 'girl*' OR ‘AYA’ OR ‘young people’ OR ‘young person*’
AND
‘sexual reproductive health’ OR ‘sexual health’ OR ‘sexual right*’ OR ‘sexual reproductive health right*’ OR ‘SRH’ OR ‘ASRH’ OR ‘SRHR’ OR ‘ASRHR’
AND
‘engagement’ OR ‘participation’ OR ‘empowerment’ OR ‘activism’ OR ‘digital activism’ OR ‘digital network*’ OR ‘participatory approaches’ OR ‘engagement strateg*’ OR ‘public representation’ OR ‘public engagement*’ OR ‘social inclusion’ OR ‘voice and agency’ OR ‘social capital’ OR ‘sense of control’ OR ‘social cohesion’ OR ‘active citizenship’ OR ‘independence’ OR ‘collective action’ OR ‘accountability’ OR ‘public space’

Annex 1 provides an overview of the search terms and the database-specific search results. We searched across disciplines by way of using Web of Science and Scopus. We discuss the selection of grey literature further below.

- Step 3: Document Selection and Appraisal

Besides rigour, the decision to include studies in realist reviews is based on the relevance to the review subject and the purpose of the review [6]. As inclusion and exclusion criteria, we retained (1) empirical studies, (2) English language studies, and (3) low- and middle- countries, and high-income countries. We included conference papers, but did not include comments without empirical content, nor did we include systematic reviews.

We used the WHO definition of adolescents, which is also followed by UNICEF and which represents the age range from 10 to 19 years [24]. We included mothers to capture sexual and reproductive health programmes that focus on pregnant teenagers and teen mothers. Finally, we included interventions focusing on mental health as adolescent well-being is also an important aspect of sexual and reproductive health in adolescents’ transition phase, for example in relation to sexual violence towards adolescents [32].

The selection and appraisal of the studies were discussed by the review team in regular meetings during the period from April to June 2021. We tried to be as inclusive as possible so as not to exclude studies that are rich in data or reporting on context, even if they show methodological weaknesses, for example in terms of reporting on data collection processes or analysis.

The selection and appraisal process was iterative. In order not to miss data on implementation, including the lessons learnt from the different ways the topic of digital empowerment was translated through programme designers’ assumptions, we broadened our selection to include interventions related to the concept of “digital literacy”.

We started the search process with a database search of Embase (v.6.2), Web of Science, and Scopus, accessed through the library of the University of Ghent, Belgium, between April and May 2021. The search extended beyond traditional databases to include grey literature as not all recent programme evaluations are published in peer reviewed journals. Similarly, non-health journals were also included in the search. Through snowballing, berry picking, and citation tracking, we came across relevant 83 publications including grey literature. The grey literature mainly come from evaluations of programmes of international NGOs.

**Fig. 2:**
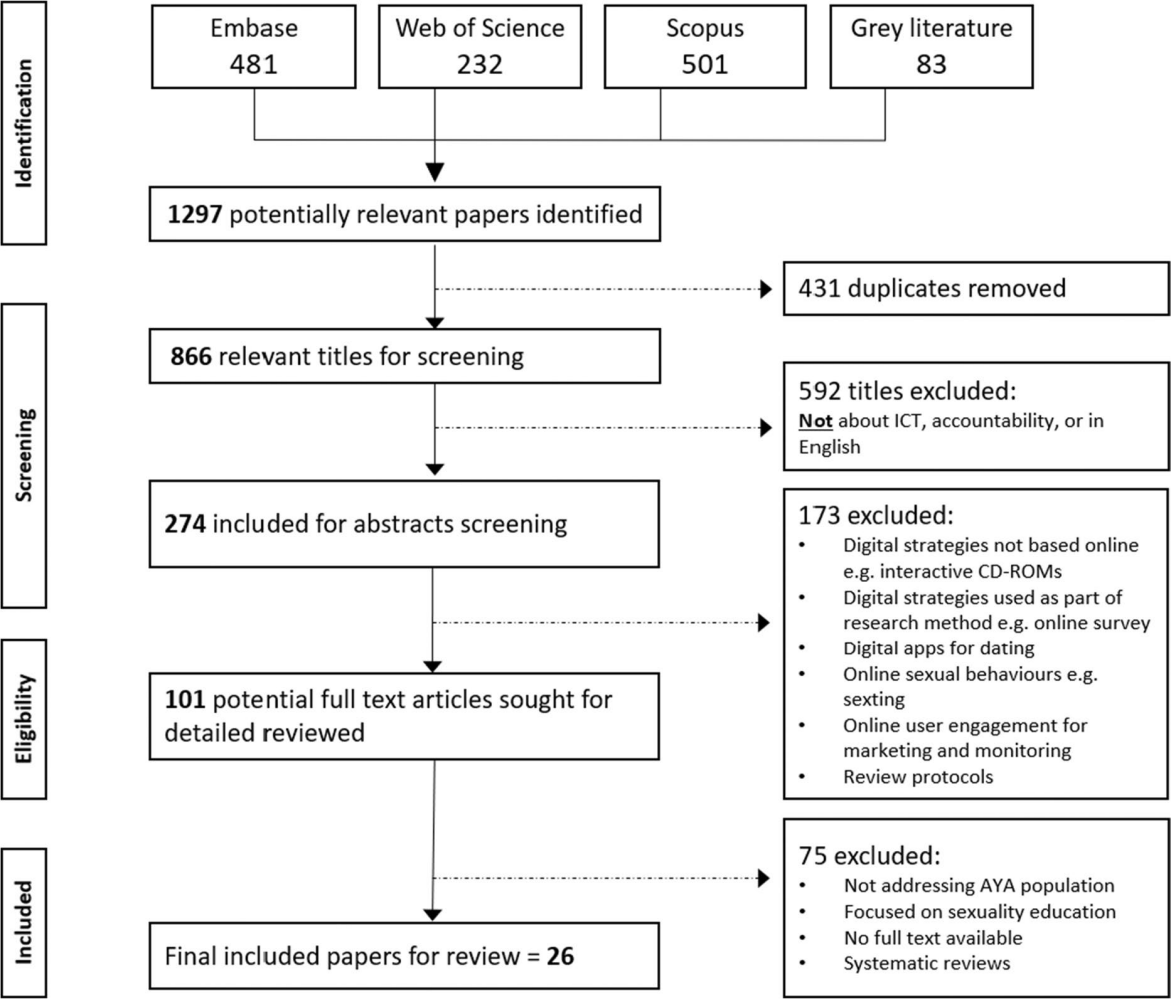
PRISMA flowchart

We identified a broad range of articles from our four sources (Fig. 2)—1297 potentially relevant papers were identified with 431 duplicates removed. Eight hundred sixty-six relevant titles were screened, and 592 excluded because they were not relevant (not about ICT, adolescents, empowerment, or accountability); the abstracts were screened of 274 articles, and 173 were excluded as not being related to being a protocol for research or online surveys for the sake of research, studies related to online sexting or dating app behaviour, or apps for dating or marketing. From the 101 articles that were reviewed in detail, 26 were selected.

We reviewed the study’s characteristics, study design, digital strategies mentioned, and its main findings (See also Annex 2). Of the 26 papers, 16 are on digital strategies in LMIC [23, 33–47]. Five papers related specifically to urban settings [34, 43, 48–50], of which one on informal settlements [42]. Sub-groups of adolescents and young adults are the target group; seven papers focused on young girls and young women [35–37, 44, 48, 49] one on young men [50], two on college student [51–53], and two on young black gay, bisexual, and men who have sex with men [34, 48]. Twelve out of 26 publications reported on digital interventions with outcomes related to sexual and reproductive health and rights. These papers focused on the access to information as the gateway to behaviour change and improved SRHR norms [33, 34, 36, 38–40, 42, 43, 48, 50, 54, 55].

A broad range of type of studies was included in this review. Three were evaluations [37, 38, 50], and one study was a randomized controlled trial [49]. Nine publications were qualitative research studies, including a qualitative survey [51], case studies [43–45], an ethnography [36], and a discourse analysis [48].

Finally, four studies were reports describing interventions [34, 35, 40, 41], while two papers presented literature reviews [23, 56]. Four publications were commentaries with empirical content [39, 54, 57, 58].

One publication focused on rural communities in India, and one targeted mothers. Moitra et al. reported on the use of mobile platforms in rural zones in India [41] and Gogoi et al. reported on an intervention for young mothers to report on quality of care [45]. We also included publications with a range of outcomes other than SRHR. For example, Sakil discussed the role of ICT in strengthening adolescents’ voice and democratic space in Bangladesh [51]. Crone et al. identified principles for putting accountability into action via digital platforms to achieve engagement and building leadership of women and girls [59], while Waldman & Stevens (2015) reported on a mixed media intervention for adolescents addressing a broader remit of issues including collective empowerment and challenges at health systems level [47].

A plethora of digital strategies and instruments were reported: use of Twitter [57], WhatsApp groups and online forums [34, 48], tele-counselling, and anonymous Q&A through text messaging [33]. We also found text messaging to push content on sexual and reproductive health [43], on geographically related access to products and services or providing information regarding the safety of public spaces [44]. Other techniques include the use of digital billboards to deliver sexual and reproductive health and rights information [42], web-based solutions for schools [49], a crowd-mapping app to inform young women on the safety of public spaces [44], and quality of care assessment through mobile phone apps [56]. Three studies mention participatory approaches to the design of the instrument or the intervention [35, 43, 54]. There is a pattern of using digital interventions within mixed media strategies, such as in the paper of Waldman & Stevens, where television series and radio were used concurrently with social media [47]. We also found examples of combinations of mobile with online platforms [60].

It appears that the main rationales behind the programmes are (1) to create a safe space for engagement on sexual and reproductive health and rights issues, and (2) to increase access to SRH information and services. Examples include the papers by Rockiki & Fink [38] and Tallarico et al. [40]

- Step 4: Data Extraction in the Form of CMO Mapping

In contrast with Hunter et al., we used the CMO heuristic also for the data extraction. We structured our data extraction form around the ICAMO configuration, a variant described by Marchal and colleagues [61]. For each paper included in the review, we listed the intervention, the actors involved, and for each strategy the enabling or “disabling” context conditions, the mechanisms triggered in these context conditions and the outcomes. The concept of mechanism is still widely debated in realist circles. We adopted the definition presented by Van Belle and colleagues: “(…) “mechanism" in the realist sense does not equate to intervention components but rather to how the resources and opportunities created by the intervention are taken up (or not) by people in different contexts.” The authors continue: “Mechanisms can play out at the level of individuals, groups, organisations and society. Ideas about mechanisms can be found in psychological, social, cultural, political and economic theories” [62]. As mentioned in our “rough” IPT, the outcome of interest is improved SRHR outcomes and well-being for adolescents in resource-constrained settings.

Realists use retroduction during the analysis, which combines deduction and induction to look and identify the underlying mechanisms that explain the outcomes [63]. In our study, step one allowed us to identify concepts and theories that pointed to mechanisms. In the data extraction step, we searched for mechanisms within the papers, guided but not restricted by our rough IPT. The evidence on the basis of which we identified mechanisms is found in Annex 3.

Our extraction process showed that, as can be expected when reviewing a body of literature with diverse designs, few papers detailed the context conditions in which the intervention took place. Also mechanisms were often not explicitly discussed: programme impact or effectiveness evaluations do not search for mechanisms and are not geared towards theory building across programmes or settings, as they mainly seek to answer the question “does it work?” [64]. This is where the deductive element kicks in, providing hunches and ideas about what may have caused the observed results, and evidence of which we looked for across the papers. “Retroduction uses both inductive and deductive logic, as well as insights or hunches. It involves thinking through what causal powers might be at work in producing observed patterns or changes in patterns” [65].

We present a summary of the key CMO configurations below in Table 3. A full overview of extracted CMO configurations from the reviewed literature can be found in Annex 3.

**Table 3:** CMO configurations contributing to refining the initial programme theory

Theme	Key CMO configurations contributing to refined programme theory	References
Enlarged spaces of engagement and dialogue	**[Intervention]** Integrating appropriate new media technologies **[Triggered Mechanisms]** *Enlarged virtual communication spaces* **[Outcomes]** Youth engagement, SRHR dialogues with multiple actors	Awan Usmail & Eboi, 2020
	**[Enabling conditions]** Peer-level support online; affiliation **[Triggered Mechanisms]** *Sense of connection; self-affirmation* **[Outcomes]** Increased resilience, rejection of judgmental or stigmatizing views	Barry et al., 2018
Anonymity and confidentiality	**[Intervention]** Mobile services offer anonymity and competition (*game*) **[Triggered Mechanisms]** *Sense of safety (not being judged*); empowerment, engagement *(*via* extrinsic motivation*), *self-determination* **[Outcomes]** extended beyond schools and community centres – wider reach	NCCE, 2011
	**[Enabling conditions]** Anonymous text service, safe space, age-appropriate content **[Triggered Mechanisms]** *Sense of security and privacy, relevance* **[Outcomes]** Engagement of youth with knowledge and information about local sexual reproductive health and rights services	Merrill, 2013
	**[Intervention]** Safe space, facilitated peer support **[Triggered Mechanisms]** *Sense of connection; self-affirmation* **[Outcomes]** Explore opinions, feelings, and prejudices in dialogue with their peers (voice)	Hightow-Wiedman et al., 2015
Access to information	**[Intervention]** Wide availability, dissemination of information **[Triggered Mechanisms]** *Sense of safety* **[Outcomes]** Informed decision making, improvements to young people’s SRH	Guerrero et al., 2020
Adapted digital information and services	**[Intervention]** language adaptation to youth, content adaptation to gender, specificity of messages **[Triggered Mechanisms]** *Ease of understanding, Competence* **[Outcomes]** Increased knowledge and making informed decisions for SHR/R; better able to provide feedback and opinions	Guerrero et al., 2020
Policy choices	**[Intervention]** Reduced state control of information dissemination **[Triggered Mechanisms]** *Active engagement of youth with online content, increasingly diverse voices* **[Outcomes]** Changes in how young people access online SRHR information, and what content is created and shared online	Hildebrand et al., 2013

During our analysis, we made some cross-cutting observations. First, there is evidently an evolution in the use of ICT—its development has been fast paced in the last decade. Some of the early publications, from 2010 until 2015, reflect programmes’ initial restricted focus on the use of ICT for life skills education [33].

Second, across interventions, we see that change is often still considered at the level of individual behaviour change. There is little consideration of the meso- or macro-level, i.e. group-level change, organizational change, or change at the level of the health system or health policy [34, 43, 50]. In contrast, and, to an extent, the paper by Tanner addressed collective action for change [44]. In addition, in most interventions and studies, there appears to be hardly any reporting on differentiation of strategies for early, late, or middle-age adolescents, nor for young adults, despite there being differentiation of strategies in terms of gender, such as in the report of Kaleidos Research [37].

A third observation is that there is no information regarding how specific context conditions interact with programme components. Only one intervention reported on the fundamental difference between urban and rural contexts [47]. If enabling or impeding context conditions are reported, it is at a descriptive level. Sociocultural norms and the political context (including for example political will) are mostly described as implementation barriers impeding effectiveness. Context is also mentioned implicitly as “programme implementation challenges,” such as the lack of stable internet in urban informal settlements in Kenya and difficulties in reaching marginalized girls in Kampala [19].

Fourth, actors are often not focused on. For example, in relation to the paper detailing the use of a crowd-mapping tool as a technology to increase safety of the urban environment for adolescent girls in Delhi, Kampala, and Lima, we do not know what the role and engagement of the authorities are, and how the authorities impede or enable the intervention [54].

Fifth, realists are interested not only in positive outcomes, but also in negative or unexpected outcomes. We found these are rarely reported. Some papers include a discussion of counterproductive outcomes, such as the risks involved with interaction with social media such as cyber-bullying, cyber-violence, and profiling [55, 56]. Banaji et al. caution against over-enthusiasm for the effects of the use of digital technology as these interact with, for example, gatekeepers which constrain girls’ access to the digital world to curb their agency [23]. A final observation is that co-production of digital technologies appears to have been applied as a programme strategy in only three interventions [54, 66, 67].

- Step 5: from Data Synthesis to Refined Programme Theory

We moved from the ICAMO configurations to the programme theory by using the following techniques detailed in Hunter et al. in discussion meetings of the review team. We used “consolidation” where we brought together different sources agreeing on similar mechanisms and outcomes, leading to the “if -then” clauses below. We defined the outlier clauses below, for which there was scant evidence. We also used the technique of “situating” and “reconciling”, where we formulated plausible explanations for the outcomes. Finally, one team member wrote up the “if–then / because” statements of the refined programme theory, which all team members subsequently reviewed and agreed upon. We also agreed upon outlier clauses, for which there is evidence in the review. We also formulated rival clauses, which are in opposition to the refined programme theory, for which there is scant evidence in the review [16].

The refined programme theory is formulated as follows:

- **IF** digital technologies provide (1) enlarged spaces of engagement and dialogue between **adolescents**; (2) access to SRH information for adolescents, even for those services that are illegal; and (3) anonymity and confidentiality, **THEN** this leads to increased engagement with digital SRH and SRHR information **BECAUSE** of a *sense of safety (*not being judged) and access to SRH information on adolescents’ “own terms”*.*
- **IF** adolescents have access to information and services adapted to their needs through digital technologies, **THEN** they will be able to make an informed decision regarding their sexual and reproductive health and well-being and to demand better care, leading to an individual ‘s improved sexual and reproductive health and rights outcomes **BECAUSE** of a better understanding of SRH and SRHR issues through increased *individual agency and self-determination.*

Outlier clauses, for which there was scant evidence in the review and which indicate gaps in current evidence, include:

- If adolescents have access to information and services adapted to their needs through digital technologies, then this will lead to emergent collective action and demand for accountability and more inclusive decision-making (a seat at the table) leading to a challenge to the status quo and social change.
- Co-production of digital technologies will produce a digital engagement space with a diversity of perspectives and enhanced ownership among, leading to adolescents’ empowerment and self-determination.
- Production of digital applications and content needs to be tailored to the setting in which the adolescents reside (e.g. rural versus urban context, urban informal settlements or poor neighbourhoods) and the adolescent subgroup (e.g. degree of marginalisation, current use of social media).

Rival clauses, which are in opposition to clauses of the refined programme theory above, include (Table 4):

- Digital technologies are shaped by the prevailing culture and gender norms, and reflect dominant social hierarchies, potentially perpetuating these.
- Digital technologies and connectivity are proprietary; they depend on an internet provider providing services to a poor neighbourhood or access to ICT devices, owned by multiple people or a household in poor settings.
- Digital technologies can be traced; digital technology is increasingly used by states for **surveillance** of communities and specific groups, marginalized groups for example men having sex with men might be subject to surveillance or have their privacy compromised.

**Table 4:** The refined programme theory

	IF	THEN	BECAUSE
Refined programme theory	**IF** digital technologies provide (1) enlarged spaces of engagement and dialogue between **adolescents**; (2) access to SRH information for adolescents, even for those services that are illegal; and (3) anonymity and confidentiality, **THEN** this leads to increased engagement with digital SRH and SRHR information **BECAUSE** of a *sense of safety* (not being judged) and access to SRH information on adolescents’ “own terms”		
	**IF** adolescents have access to information and services adapted to their needs through digital technologies, **THEN** they will be able to make an informed decision regarding their sexual and reproductive health and well-being and to demand better care, leading to an individual ‘s improved sexual and reproductive health and rights outcomes **BECAUSE** of a better understanding of SRH and SRHR issues through increased *individual agency and self-determination*		
**Outlier clauses**, for which there was scant evidence in the review, and which indicate gaps in current evidence	If adolescents have access to information and services adapted to their needs through digital technologies, then this will lead to emergent collective action and demand for accountability and more inclusive decision-making (a seat at the table) leading to a challenge to the status quo and social change		
	Co-production of digital technologies will produce a digital engagement space with a diversity of perspectives and enhanced ownership among, leading to adolescents’ empowerment and self-determination		
	Production of digital technologies and related content needs to be tailored according to the setting in which the adolescents reside and their subgroup characteristics. These include rural or urban contexts; urban informal settlements or poor neighbourhoods; age; whether the target group is marginalized and have limited access to digital technologies; and which social media are already in use		
**Rival clauses**, which are in opposition to clauses of the refined programme theory above	Digital technologies are shaped by the prevailing culture and gender norms, and reflect dominant social hierarchies, potentially perpetuating these		
	Digital technologies and connectivity are proprietary; they depend on an internet provider providing services to a poor neighbourhood or access to ICT devices, owned by multiple people or a household in poor settings		
	Digital technologies can be traced; digital technology is increasingly used by states for **surveillance** of communities and specific groups, marginalized groups for example men having sex with men might be subject to surveillance or have their privacy compromised		

#### Zooming in on the Mechanisms

One of the mechanisms underlying the use of digital technologies is that it creates a “safe space”, where adolescents in general, and marginalized groups more specifically, do not feel judged, as it is confidential, and anonymous at best [34]. In this way, the digital world appears to work as a “shield” against gatekeepers (parents, providers), prevailing socio-cultural norms, stigma and discrimination. They enhance adolescents’ access to information on services, directions to youth-friendly clinics, information on laws, and practices that are sociocultural taboos or are illegal in certain settings, such as abortion [53, 59, 60].

Second, solidarity between adolescents going through the same issues, by way of sharing of stories, dialogue, and engagement between peers seems to be a mechanism programme’s aim to trigger, because this is considered to foster resilience [48], a sense of connection [34, 44, 48], and because of adolescents’ distrust of providers and institutions in settings with a dominant conservative tradition [43].

Third, digital technologies are user-oriented; they can be accessed on adolescents’ own terms, i.e. in a space and at a time which is convenient for the user [33]. The latter process is captured by Eysenbach’s concepts of disintermediation and apomediation [68]. Disintermediation refers to the process where digital technologies enable users to access information without mediation and thus to navigate around gatekeepers who may actively curb access. Apomediation is the mechanism which indicates the shift from access to credible info through experts as gatekeepers to social networks online, and the role of online peer influencers. There has been quite a limited view on the role different actors play in interventions, and the interaction between the intervention and apomediation where influencers and exchange between peers play a dominant role in access to information should be taken into account in intervention design [52, 69].

#### Zooming in on the Actors Besides the Adolescents

Programmes differ according to the setting in terms of socio-cultural norms and the role of state actors. Progressive governments tend to sponsor the development of the tool. In other programmes, local government authorities, the police, or policy makers are considered as targets for advocacy, or groups who should be held to account through the data generated by the programmes [39]. In addition, state actor interests to be engaged in digital technology might differ significantly from adolescents’ needs.

Parents are in mainly considered as gatekeepers constraining access to sexual and reproductive health information, and there is a difference between parents’ knowledge of digital technology and what adolescents actually can do [23, 44]. In many programmes, the role of parents is a passive one or they may have a negative connotation. In the same vein, school teachers and health providers appear to be mainly gatekeepers, curbing access to information, and being harbingers of shame and stigma. Mentioned above, in the design of digital technology, we should consider also the shift from ASRH knowledge to the role of peer influencers in accessing trustworthy information [69].

- Step 6: Dissemination of Findings

In this step, we formulated recommendations on the based on our realist-informed review. They may serve as a guide and reminder for policy-makers and practitioners commissioning, formulating, implementing, or evaluating future digital interventions in the context of adolescents SRHR programmes in urban settings, as well as researchers:

- Production of digital technologies and content needs to be **tailored** to the **setting** in which the adolescents reside and needs of the adolescents.
- **Co-production** of digital technologies may contribute to digital engagement spaces where a diversity of perspectives is present and to enhanced ownership, which in turn may lead to empowerment and self-determination.
- **Disintermediation and emergent apomediation** may be powerful mechanisms, mediating the roles of different actors (state actors, teachers, parents, health providers) as well as the adolescents. They may trigger a shift towards “standing by” instead of “standing in between” adolescents and sexual and reproductive health and rights information.

The findings of this study will also inform further research. They will be used to complement other reviews on urban health governance in LMIC and a systematic review on accountability interventions in sexual and reproductive health in LMIC in the Msichana/Chakti ! [70]. Furthermore, members of the review team are part of communities of practice, such as the Community of Practitioners on Accountability in Health (www.COPASAH.org) and the Community of Practice on accountability in health hosted by WHO Department of Reproductive Health. In these CoPs, this review will be presented and discussed with key stakeholders and further dissemination will be facilitated by member practitioners by way of their networks including affiliate NGOs and researchers in LMIC.

## Conclusion

Our review identified two gaps in the research on digital interventions for urban adolescents. Both relate to a seeming under-theoretisation of certain concepts the programmes rely on in implementation. The first pertains to the urban environment the programme has to operate in and its meaning for the adolescents. We note in this area the absence of a “southern” urbanist perspective. Unless there is a co-production strategy to the intervention, we noted in the publications that there is no specific view or contextual specificity regarding what the “urban” consists of for adolescents living in LMIC, what upstream determinants are involved, and the huge variety which falls under its flag.

Second, the socio-developmental stage of the adolescents the programmes work in is often ignored. In the current literature, there is virtually no reference to theories related to socio-cognitive, moral, or psychosocial development of adolescents (such as Kohlberg, Eriksen, or Piaget) and how strategies are modified according to developmental age. Further empirical theory-building and theory-testing is a research priority in this field.

## Supplementary Information

Below is the link to the electronic supplementary material.

Supplementary file1 (DOCX 262 KB)

Supplementary file2 (DOCX 44.7 KB)

Supplementary file3 (DOCX 794 KB)
