# Supplementary material for: A Realist-Informed Review of Digital Empowerment Strategies for Adolescents to Improve Their Sexual and Reproductive Health and Well-being
Source: J Urban Health. 2022 Sep 7;99(6):1141–56. doi: 10.1007/s11524-022-00678-8 (PMC9727007; doi:10.1007/s11524-022-00678-8)
Supplement: Supplementary file 1 — Supplementary file1 (DOCX 262 KB) [file 11524_2022_678_MOESM1_ESM.docx]

Search log

Major concepts

| ICT strategies | AND | Adolescents | AND | ASRH + ASRHR | AND | Accountability |
| --- | --- | --- | --- | --- | --- | --- |

Initial search terms

| Digital technology  Mobile phone Cell phone use  Web-based intervention* Social media ICT  Internet  Digital media  DCT  Online | AND | Adolescent*  Youth  Young adult* Girl*  AYA  Young people  Young person* | AND | Sexual reproductive health  SRH  Sexual health  Sexual right*  Sexual reproductive health right*  SRHR | AND | Accountability  Engagement  Participation  Empowerment  Activism  Digital activism  Digital network*  Participatory approaches  Engagement strateg*  Public engagement  Public representation  Social inclusion  Voice and agency  Social capital  Sense of control  Social cohesion  Collective action  Active citizenship  Independence |
| --- | --- | --- | --- | --- | --- | --- |

# Database searching: Embase

|  | Search strategy | Results | Comments |
| --- | --- | --- | --- |
| Date:  9/3/21 | 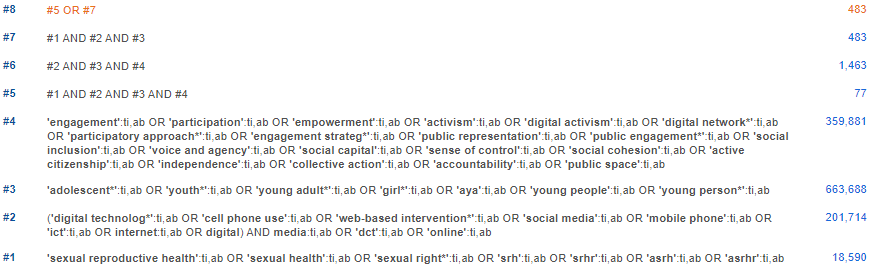 | 483 – 2 duplicates =  481 | #5 all included in #8, therefore keeping a larger result set for screening to include less obvious but relevant papers |

Database searching: Web of Science

|  | Search strategy | Results | Comments |
| --- | --- | --- | --- |
| Date: 8/3/21 | 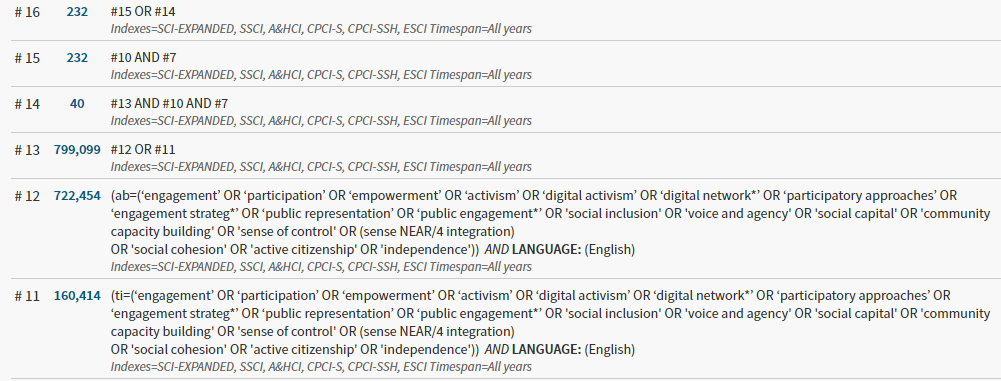  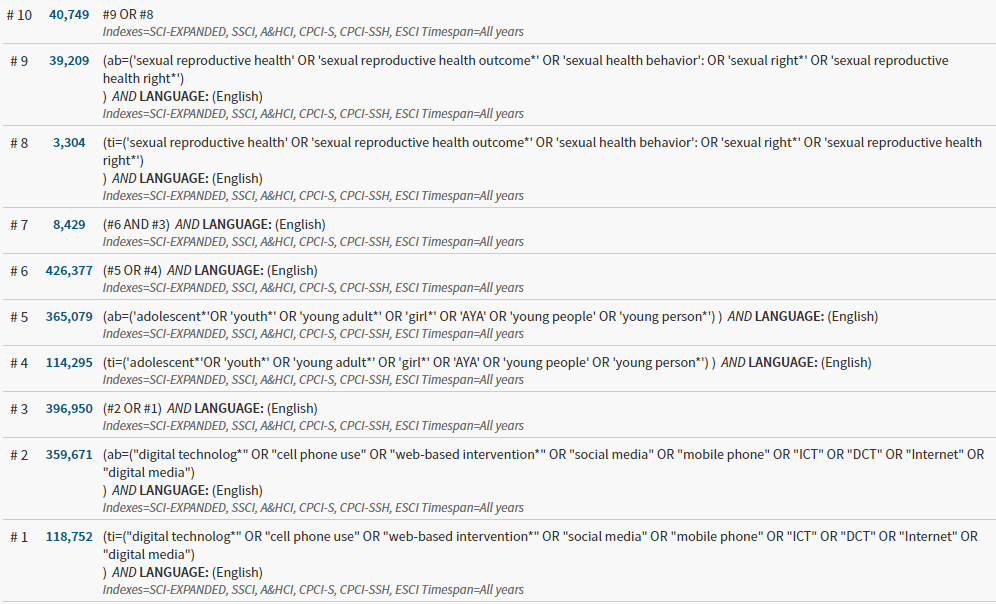 | 232 |  |

Database searching: Scopus

|  | Search strategy | Results | Comments |
| --- | --- | --- | --- |
| Date: 8/3/21 | TITLE-ABS-KEY ( ( adolescent* ) OR ( youth* ) OR ( young AND adult* ) OR ( girl* ) OR ( aya ) OR ( young AND people ) OR ( young AND person* ) ) AND TITLE-ABS-KEY ( ( digital AND technolog* ) OR ( cell AND phone AND use ) OR ( web-based AND intervention ) OR ( social AND media ) OR ( mobile AND phone ) OR ( ict ) OR ( internet ) OR ( digital AND media ) OR ( dct ) ) AND TITLE-ABS-KEY ( ( sexual AND reproductive AND health ) OR ( sexual AND reproductive AND health AND outcome ) OR ( sexual AND health AND behavior ) OR ( sexual AND right* ) OR ( sexual AND reproductive AND health AND right* ) ) AND TITLE-ABS-KEY ( ( engagement ) OR ( participation ) OR ( empowerment ) OR ( activism ) OR ( digital AND activism ) OR ( digital AND network* ) OR ( participatory AND approaches ) OR ( engagement AND strateg* ) OR ( public AND representation ) OR ( public AND engagement* ) OR ( social AND inclusion ) OR ( voice AND agency ) OR ( social AND capital ) OR ( sense AND of AND control ) OR ( sense AND near/4 AND integration ) OR ( social AND cohesion ) OR ( active AND citizenship ) OR ( independence ) ) ) | 501 |  |
