# Supplementary material for: A Realist-Informed Review of Digital Empowerment Strategies for Adolescents to Improve Their Sexual and Reproductive Health and Well-being
Source: J Urban Health. 2022 Sep 7;99(6):1141–56. doi: 10.1007/s11524-022-00678-8 (PMC9727007; doi:10.1007/s11524-022-00678-8)
Supplement: Supplementary file 2 — Supplementary file2 (DOCX 44.7 KB) [file 11524_2022_678_MOESM2_ESM.docx]

| **Table 2**. **Characteristics and findings of papers meeting review criteria (n=26)** | | | | |
| --- | --- | --- | --- | --- |
| **Reference** | **Country / Setting** | **Study design** | **Digital  Intervention / Strategy** | **Main findings** |
| 1_Crone et al. (2018)  **#WhatWomenWant: A new accountability**  **paradigm and expanded accountability**  **mechanisms through leveraging social media,**  **catalysing movements, and building leadership.** | Global  Women and girls | Policy brief | #WhatWomenWant consulting platform:   Ongoing Whatsapp-facilitated focus group, spaces for engagement | Identified principles for putting accountability into action:  -Act on commitment  -Consult widely, constantly  -Women and girls leadership  -Engagement of most affected  -Making information accessible  -inform women about commitments of governments  -Network building |
| 2_NCCE (2011)  **Learning About Living – Nigeria Project Final report.** | Lagos, Cross River& Abuja (Nigeria)  Youth | Program evaluation | Learning about Living (LaL) project.   E-learning system with associated mobile component for anonymous question and answer service. | Improved discussion and information on reproductive health education and dampened effects of socio-cultural tendencies that lead to a reluctance to discuss these issues amongst young people  Increased gender equality by reducing the prevalence of and offering positive alternatives to gender constructs that assume male superiority and the acceptability of violence against women in Nigerian society |
| 3_Awan Ismail & Eboi. (2020)  **New Media Strategies’ Model for Sexual Reproductive Health & Rights Campaigns among Young People in Informal Settlements: Mitigating the Challenges.** | Kenya;  Informal settlements  Young people | Qualitative  Focus groups discussions   In-depth interviews with stakeholders | New media strategies:  Key messaging   School-web-based solutions   Combination strategy  New media-geographic-based strategy  Digital billboards   Sustained solo strategy | Challenges identified:  - Opposition: online group of parents actively oppose SRHR-related issues  - Messages: Failure to control of message on platforms and timing; unengaging messages  - Limited finance and resources of AYA and media managers  - Technology: Electricity issues and availability of stable internet. Training media managers  - Implementation: Some young people are not trained in SRHR issues but are very fast in churning out content. Others have SRHR training and experience but may be incompetent. |
| 4_Guerrero et al. (2020)  **Developing an SMS text message intervention on sexual and reproductive health with adolescents and youth in Peru.** | Lima, Ayachucho & Loreto (Peru)  Urban and peri-urban districts  Adolescents | Case study on intervention content development    Participatory approach with AYA | Private SMS platform for sending and receiving SRH information | Differences in SRH/SRHR information needs between adolescents (13-17) and youth (18-24)  Recommendations:  Language used should be simple. Increase relevance by appealing to adolescents personal experiences.  Importance of including information about violence:  what constitutes abuse and violence; reinforce information about individual’s rights and value as a person |
| 5_Nanda et al. (2020)  **“The Times They Are A-Changin”: using technology for ASRHR in the 25 years since ICPD.** | Online  Covid-19  Adolescents | Commentary with empirical content | Tele-counselling through AR/VR and gamification  Multi-media content for behavioural and norms changes   Tech-enabled and geo-tagged access to services and products | Caution for technology use:  - Broadly accessed technology may become a tool for surveillance, censorship, curbing dissent, and targeted discrimination and harm.  - Technology interacts with social, cultural, and political aspects, intersecting with laws and policies that may be uniquely restrictive of adolescents’ SRHR  - Risks of cyber violence, bullying, profiling for younger age groups  Need for:  Inclusive voices and digital literacy |
| 6_Barry et al. (2018)  **“Stay strong! keep ya head up! move on! it gets better!!!!”: resilience processes in the healthMpowerment online intervention of young black gay**, **bisexual and other men who have sex with men.** | Southeast United States  YBMSM | Discourse analysis of conversations using the resilience framework (qualitative research) | HealthMpowerment.org (HMP) :   An internet and mobile phone-based intervention consisting of an anonymous online space and forum facilitating conversations | Four primary resilience processes identified:  - Exchanging social support  - Engaging in health-promoting cognitive processes  - Enacting healthy behavioral practices  - Empowering others |
| 7_Hightow-Weidman et al. (2015)  **HealthMpowerment.org: building community through a mobile-optimized, online health promotion intervention.** | North Carolina, United States  YBMSM | Intervention pilot study/trial without concurrent controls + CASI survey | HealthMpowerment.org (HMP) :   An internet and mobile phone-based intervention consisting of an anonymous online space and forum facilitating conversations | HMP demonstrated acceptability and feasibility  Improvements on key mental health and social support indicators |
| 8_Rokicki & Fink (2017)  **Assessing the reach and effectiveness of mHealth: evidence from a reproductive health program for adolescent girls in Ghana.** | Accra, Ghana  Adolescent girls | Evaluation of program effectiveness  Secondary data analysis | Interactive mobile phone quiz | mHealth programs for adolescents have the potential to engage and increase SRH knowledge of adolescent girls across sociodemographic strata, including those who may be at higher risk of poor SRH outcomes. |
| 9_Hildebrand et al. (2013)  **CrowdOutAIDS: crowdsourcing youth perspectives for action.** | Global, online forums  Youth | Comment with secondary data analysis | CrowdOutAIDS: participatory online policy project | Through leveraging social media and crowdsourcing, it is possible to integrate grassroots perspectives from across the globe into a new model of engagement and participation, which should be further explored for community empowerment and mobilization. |
| 10_Merrill et al. (2013)  **Pretesting an mHealth intervention for at-risk adolescent girls in Soweto, South** **Africa: Studying the additive effects of SMSs on improving sexual reproductive health & rights outcomes.** | Soweto, South Africa  Adolescent girls | Intervention test and refine  Participatory process | SMS campaign:  Coach Tumi prototype with messaging USSD line | Results from the Coach Tumi prototype suggest that a USSD line offers a promising means of reaching adolescent girls ages 11-14 with important messages pertaining to sensitive health issues and access of services.  This finding reinforces the benefit of the USSD line in allowing a girl to access the messages on any phone—not only a phone belonging to her. |
| 11_Tallarico et al. (2018)  **TUNE ME: A mHealth initiative to increase young people’s knowledge and skills to promote the adoption of protective sexual behaviours.** | South Africa  Young people | Report | Tune Me mHealth initiative:   Features include geo-location and feedback mechanisms to track quality of care  Campaigns via Facebook, radio and mobile web banner | TuneMe is well underway to become the ‘go to’ mobisite for reliable, realistic, and holistic sexual and reproductive health information in the region.  TuneMe’s long-term goal is to empower local youth groups to take ownership of the content, learn to run its mobisite and Facebook platform, and use them as advocacy tools in campaign activities. |
| 12_Herbst (2017)  **# ByeTaboo: Expanding Access to Sexual and Reproductive Health and Rights Education.** | Buenos Aires, Argentina  Young people | Comment with empirical content | Online platform #ChauTabú (#ByeTaboo)  Strategy tackled taboos and sought to include rights-based and non-heteronormative approaches | Online platforms can offer innovative and interactive ways of delivering information on sexuality  #ChauTabú fills a much-needed gap in Argentina – especially for women |
| 13_Gannon et al. (2020)  **A mobile sexual health app on empowerment, education, and prevention for young adult men (MyPEEPS mobile): acceptability and usability evaluation.** | Chicago and New York, United States  Young adult men | Evaluation  Usability questionnaires and in-depth interviews | MyPEEPS Mobile app  (Abbrev: Male Youth Pursuing Empowerment, Education and Prevention around Sexuality) | Participants reported that MyPEEPS Mobile was free of functional problems, easy to use, and useful, with an engaging approach that increased acceptability, including the use of avatars and animation, and inclusive  representation of the diverse identities by race and ethnicity, gender identity, and sexual orientation. |
| 14_Fongkaew & Fongkaew (2016)  **My space, my body, my sexual subjectivity: social media, sexual practice and parental control among teenage girls in urban Chiang Mai.** | Chiang Mai, Thailand  Teenage girls | Ethnography | Social media ‘secret groups’:  Where girl group members shared confidential stories, sexual fantasies and sexual experiences seen exclusively by group members | Online social networks play an important role by providing an alternative cyberspace  for young women to gather together through online social networks. Through social media utilisation, young  women are able to assert their agency and find ways to negotiate and challenge traditional forms of femininity. |
| 15_Widman et al. (2018)  **Sexual assertiveness skills and sexual decision-making in adolescent girls: randomized controlled trial of an online program.** | Southeastern United States  Adolescent girls | Program evaluation  Randomised controlled trial | Interactive, web-based sexual health  program  (Health Education and Relationship Training [HEART]) program for developing sexual assertiveness skills and enhancing sexual decision-making in adolescent girls. | Girls who completed the HEART program demonstrated better sexual assertiveness skills measured with a behavioral task, higher self-reported assertiveness, intentions to communicate about sexual health, knowledge regarding HIV and other sexually transmitted diseases (STDs), safer sex norms and attitudes, and condom self-efficacy compared with the control group. |
| 16_Waldman & Stevens (2015)  **Sexual and reproductive health rights and information and communications technologies: A policy review and case study from South Africa.** | South Africa  Young people | Case study | 1 Young Africa Live (YAL) social network on mobile platform  2 Soul City – mixed media including television series, radio and social media | 1 YAL is politically progressive, seeks to discuss and challenge social norms. ICT and mobile phones are seen by YAL’s implementers as facilitating social change, because information can be personalised to users.  2 Aims to improve people’s quality of life and health; strengthening individuals, communities and society through active citizenship, human rights and social  Justice. Content addressed health systems and process  issues. |
| 17_Tanner et al. (2020)  **Disruption and Design: Crowdmapping Young Women’s Experience in Cities.** | Delhi, Kampala, Lima, Sydney  Young women | Case study on -strategy / tool refinement via online survey | ‘Free to Be Map’ Crowdmapping tool | Crowdmapping offers a unique platform for collective  action – generating a safe, anonymous space with a large  number of stories, enabling trends to emerge, and patterns to be identified across voices and experiences |
| 18_ Gogoi et al.  (2018)  **Empowering Women in India to Influence Maternal Healthcare Quality Through Mobile Phones and Crowdsourcing** | India  Women | Case studies analysis  Book chapter | Mobile Monitor for Quality of maternal Care (MoM-QC) | The platform’s simple, low-tech functionality leveraged the ease of cell phone use while giving each caller the power to “give voice” and rate the services they received.  MoM-QC improved users’ knowledge of maternal health quality and entitlements. The majority of women in the feasibility study were both able and willing to rate quality of care, and health providers and government officials were receptive to receiving women’s feedback to take follow-up action as needed. |
| 19_Kaleidos Research & ICRH (UGent)  (2016)  **Access, Services, Knowledge (ASK) – Youth Empowerment Alliance (YEA): End-of-Programme Evaluation, Synthesis Report.** | Young people (aged 10–24 years) in seven countries:  Uganda Kenya Ghana Senegal Ethiopia Pakistan Indonesia | Evaluation report via:  document analysis  Web-survey with local partners + Delphi approach  Interviews (f2f/telephone)  Workshop  Focus group | The ASK programme (Access, Services, Knowledge) targets key elements in meeting young people’s needs: information and education; youth-targeted and youth-friendly services; and raising awareness and acceptance of young people’s sexuality.  Sub-component: electronic and mobile health (e&m health) tools e.g mobile phone apps, web-based information platforms, social media and chat. | The multi-component approach of ASK consists of three elements: **demand, supply and support**, which each have their own strategy but also influence each other:  Through the provision of SRHR information, the ASK programme empowers young people to make healthy and well-informed decisions (improving knowledge, skills and self-efficacy of young people).  Community sensitization, participation and mobilization activities are implemented to create an environment that accepts adolescent SRHR and increases broad community support for sexuality education and youth-friendly SRH services. Furthermore, lobbying and advocacy activities are undertaken to facilitate the creation of policies and laws that are supportive of young people’s rights and needs |
| 20_USAID & Momentum (2021)  **The power of youth voices: How youth are holding their health systems accountable for family planning and reproductive health.** | Youth  Global | Landscape analysis Report  Desk review of peer-reviewed and grey literature  In-depth interviews with youth, academics, and representatives of implementing organizations.  Virtual consultation with youth | Digital approaches mentioned in report:  MobiSAfAIDS application for phones. Youth report on the quality and delivery of FP/RH care, which then is then fed back to the facilities and SAFAids in real-time to inform quality improvement and advocacy  SAUTIPlus app operated on phones used by peer educators and computers and tablets used by health centers to collect and monitor data on quality of service provision | Impacts to date have included a marked increase in the number of young people accessing health facilities; increased awareness among government decision-makers and facility administrators of the barriers that exist for youth to access quality services.   - No impact studies done to date |
| 21_ Thackeray & Hunter (2010)  **Empowering youth: Use of technology in advocacy to affect social change.** | Youth | Comment with empirical content | Mainstream forms of ICT categorised as:  Social Networking Sites (Applications/Wall or comments group)  Mobile phones (SMS/MMS)  Other Internet-based technologies (RSS/Twitter/Blog/ podcast) | Technology makes it easy and convenient for youth to participate. It allows for integration of advocacy into their daily lives.  Making deliberate efforts  to combine technology and youth advocacy will give youth a voice, increase their personal efficacy for participating in advocacy, and impact the social determinants that affect the health status of people in their communities and throughout the world. |
| 22_ Waldman et al. (2018)  **‘We have the internet in our hands’: Bangladeshi college students’ use of ICTs for health information.** | Youth, college students  Bangladesh | Qualitative survey  Focus groups  In-depth interviews | Mobile phones and internet use | Personal searches for SRH and the resultant online information shared through discrete, personal face-to- face discussions has some potential to challenge social norms. This is particularly so for women students, as sharing information may enable them to bypass gatekeepers and make decisions about reproduction. |
| 23_Banaji et al. (2018)  **Instrumentalising the digital: findings from a rapid evidence review of development interventions to support adolescents’ engagement with ICTs in low and middle income countries.** | Adolescents  LMIC | Rapid review | ICT and digital media in general | Maximising the positive potential of ICTs requires a grounded understanding of how technology interacts with social, political, and economic factors important for development  ICTs are often intended and even assumed to act as an enabler or facilitator of equity and participation: but often they do not. Given the paucity of evidence about adolescents’ digital media uses between and among regions in LMICs, international development policy informing programmes targeting  adolescent capability development need to be critical and cautious in how ICTs are harnessed to promote child rights. |
| 24_ Huq et al. (2020)  **New forms of adolescent voice and agency through ICT and mobile phone use.** | Bangladesh  Adolescents | Policy Brief | Mobile phone and Internet | Divides in the ownership of digital devices and in internet access exist across class, location, and gender among adolescents.  All adolescents have some access to mobile phones, but the quality of access varies.  Gatekeepers’ negative assumptions might widen the  digital divide and restrict voice and agency among female  adolescents.  Access to the internet contributes to adolescent voice and building confidence.  Gatekeepers’ have an important role in building safe  environments for adolescents’ mobile and internet use. |
| 25_ Sakil (2018)  **ICT, youth and urban governance in developing countries: Bangladesh perspective.** | Youth, college students  Bangladesh | Qualitative survey | ICT and mobile platforms | ICT is not the answer to developing democratic institutions for youth in the developing world but represent an entire new channel of communication that may contribute to this broader goal.  Governance itself is transformed by fast-moving changes of ICT in the hands of the young.  Young people are developing mobile phone applications in large numbers that affect many areas of local governance and community life such as leadership and inter-governmental relations. |
| 26_Moitra et al. (2016)  **Design Lessons from Creating a Mobile-based Community Media Platform in Rural India** | Rural communities  India | Report | IVR (Interactive Voice Response) systems  Mobile phones | Content management, community mobilization and training, and institutional linkages are 3 critical non-technological functions to run a mobile-based community media platform.  These offline processes drive technology adoption, build credibility for the system in the eyes of the community, and provide a bi-directional communication conduit between the organization and its users. |

**REFERENCES**

1. Crone, T., de Graaf, K., Stevenson, J., Nyambura, C., & Johnson, E. (2018). # WhatWomenWant: A new accountability paradigm and expanded accountability mechanisms through leveraging social media, catalysing movements, and building leadership. *Agenda*, 32(1), 87-96.
2. NCCE. (2011). Learning About Living: Nigeria Project. Final report. Retrieved March 13, 2021, from http://www.ncceonline.edu.ng/download/files/LaL%20Nigeria%20project%20final%20evaluation.pdf
3. Awan Ismail, N. A. H., & Eboi, A. A. (2020). New Media Strategies’ Model for Sexual Reproductive Health & Rights Campaigns among Young People in Informal Settlements: Mitigating the Challenges. *European Journal of Molecular & Clinical Medicine*, 7(6), 1455-1473.
4. Guerrero, F., Lucar, N., Claux, M. G., Chiappe, M., Perez-Lu, J., Hindin, M. J., & Bayer, A. M. (2020). Developing an SMS text message intervention on sexual and reproductive health with adolescents and youth in Peru. *Reproductive health*, *17*(1), 1-14.
5. Nanda, P., & Tandon, S. (2019). “The Times They Are A-Changin”: using technology for ASRHR in the 25 years since ICPD. *Sexual and reproductive health matters*, 27(1), 349-351.
6. Barry, M. C., Threats, M., Blackburn, N. A., LeGrand, S., Dong, W., Pulley, D. V., Muessig, K. E. (2018). “Stay strong! keep ya head up! move on! it gets better!!!!”: resilience processes in the healthMpowerment online intervention of young black gay, bisexual and other men who have sex with men. AIDS care, 30(sup5), S27-S38.
7. Hightow-Weidman, L. B., Muessig, K. E., Pike, E. C., LeGrand, S., Baltierra, N., Rucker, A. J., & Wilson, P. (2015). HealthMpowerment. org: building community through a mobile-optimized, online health promotion intervention. *Health Education & Behavior*, *42*(4), 493-499.
8. Rokicki, S., & Fink, G. (2017). Assessing the reach and effectiveness of mHealth: evidence from a reproductive health program for adolescent girls in Ghana. *BMC Public Health*, *17*(1), 1-14.
9. Hildebrand, M., Ahumada, C., & Watson, S. (2013). CrowdOutAIDS: crowdsourcing youth perspectives for action. *Reproductive health matters*, 21(41), 57-68.
10. Merrill, J., Hershow, R., Gannett, K., & Barkley, C. (2013, December). Pretesting an mHealth intervention for at-risk adolescent girls in Soweto, South Africa: Studying the additive effects of SMSs on improving sexual reproductive health & rights outcomes. In Proceedings of the Sixth International Conference on Information and Communications Technologies and Development: Notes-Volume 2 (pp. 96-99).
11. Tallarico, R., De Beer, T., Bakaroudis, M., Samarthya-Howard, A., & Markus, H. B. (2018). TUNE ME: A mHealth initiative to increase young people’s knowledge and skills to promote the adoption of protective sexual behaviours. Journal of Development Communication, 29(1).
12. Herbst, N. (2017). # ByeTaboo: Expanding Access to Sexual and Reproductive Health and Rights Education.
13. Gannon, B., Davis, R., Kuhns, L. M., Rodriguez, R. G., Garofalo, R., & Schnall, R. (2020). A mobile sexual health app on empowerment, education, and prevention for young adult men (MyPEEPS mobile): acceptability and usability evaluation. *JMIR formative research*, 4(4), e17901.
14. Fongkaew, W., & Fongkaew, K. (2016). My space, my body, my sexual subjectivity: social media, sexual practice and parental control among teenage girls in urban Chiang Mai. *Culture, health & sexuality*, *18*(5), 597-607
15. Widman, L., Golin, C. E., Kamke, K., Burnette, J. L., & Prinstein, M. J. (2018). Sexual assertiveness skills and sexual decision-making in adolescent girls: randomized controlled trial of an online program. *American journal of public health*, 108(1), 96-102.
16. Waldman, L., & Stevens, M. (2015). Sexual and reproductive health rights and information and communications technologies: A policy review and case study from South Africa (No. IDS Evidence Report; 113). IDS.
17. Tanner, S., Kalms, N., Cull, H., Matthewson, G., & Aisenberg, A. (2020). Disruption and Design: Crowdmapping Young Women’s Experience in Cities.
18. Gogoi, A., Katoch, M., & Agrawal, P. (2018). Empowering Women in India to Influence Maternal Healthcare Quality Through Mobile Phones and Crowdsourcing. In Global Perspectives on Women's Sexual and Reproductive Health Across the Lifecourse (pp.111-123 ). Springer, Cham.
19. Kaleidos Research & ICRH (UGent). (2016). Rep. *Access, Services, Knowledge (ASK) - Youth Empowerment Alliance (YEA): End-of-Programme Evaluation, Synthesis Report*. Retrieved March 26, 2021, from <http://kaleidosresearch.nl/publication/ask-evaluation/>.
20. USAID & Momentum. (2021). Rep. *The power of youth voices: How youth are holding their health systems accountable for family planning and reproductive health.* Retrieved March 26, 2021, from <https://usaidmomentum.org/resource/youth-social-accountability-landscape/>
21. Thackeray, R., & Hunter, M. (2010). Empowering youth: Use of technology in advocacy to affect social change. *Journal of Computer-Mediated Communication*, 15(4), 575-591.
22. Waldman, L., Ahmed, T., Scott, N., Akter, S., Standing, H., & Rasheed, S. (2018). ‘We have the internet in our hands’: Bangladeshi college students’ use of ICTs for health information. *Globalization and health*, 14(1), 1-16.
23. Banaji, S., Livingstone, S., Nandi, A., & Stoilova, M. (2018). Instrumentalising the digital: findings from a rapid evidence review of development interventions to support adolescents’ engagement with ICTs in low and middle income countries. *Development in Practice*, 28(3), 432-443.
24. Huq, L., Sultan, M., & Khondoker, Z.A. (2020). *New forms of adolescent voice and agency through ICT and mobile phone use* (Policy brief). Retrieved from <https://www.gage.odi.org/publication/new-forms-of-adolescent-voice-and-agency-through-ict-and-mobile-phone-use/>
25. Sakil, A. H. (2018). ICT, youth and urban governance in developing countries: Bangladesh perspective. *International Journal of Adolescence and Youth*, 23(2), 219-234.
26. Moitra, A., Das, V., Vaani, G., Kumar, A., & Seth, A. (2016, June). Design lessons from creating a mobile-based community media platform in Rural India. In *Proceedings of the Eighth International Conference on Information and Communication Technologies and Development* (pp. 1-11).
