# Supplementary material for: A Realist-Informed Review of Digital Empowerment Strategies for Adolescents to Improve Their Sexual and Reproductive Health and Well-being
Source: J Urban Health. 2022 Sep 7;99(6):1141–56. doi: 10.1007/s11524-022-00678-8 (PMC9727007; doi:10.1007/s11524-022-00678-8)

| **Reference** | **I**_nterventions | **A**_ctors | **C**_ontexts | **M**_echanisms | **O**_utcomes |
| --- | --- | --- | --- | --- | --- |
| 1_#WhatWomenWant | Global online campaign  Consultation platform (Whatsapp-facilitated focus group) | Young women; Feminist community change agents  Peripheral, supportive  ATHENA Initiative  women’s rights activists; Feminists;  Campaigners; Advocates  Decision-makers  United Nations; World Health Organization; Governments; Ministries of Health; South African Development Community (SADC); African Union; Local level, including mayors, municipal ofﬁces, councils, and traditional leaders  Funders  European Union; Melinda Gates Fund; Global Fund | **Theme: Coordination and female Leadership (AGENCY)**  **[Enabling conditions]** coordinated (young) women-led leadership who are connected to, in conversation with, and learning together with those of us who have been around a while; vision; lead and control conversations, network building  **[Triggered Mechanisms]** Energize cause, enhanced relevance to young women, sense of control, facilitative effect  **[Outcomes]** push for change for women by women; advancing a women’s rights agenda and building a feminist movement; convene a system of tracking and accountability for young women, by young women  **Theme: Identity and representation (VOICE)**  **[Enabling conditions]** Inclusion and participation of women (ongoing ‘seat at the table’), ask women and young women for their needs, priorities, visions and solutions; clarity of women’s roles to demand accountability  **[Triggered Mechanisms]** engagement, empowerment, group effect (join forces)  **[Outcomes]** move from margins to center of engagement in SRHR  **Theme: Facilitative resources**  **[Enabling conditions]** Adapted information, social investment, funding  **[Triggered Mechanisms]** increased understanding, resource/means for expansion of movement  **[Outcomes]** make informed decisions, women engagement, transformative change, new form of agenda setting (women SRHR)  **Theme: Progressive sense of place as resource**  **[Enabling conditions]** Rapidly evolving social media landscape  **[Triggered Mechanisms]** organizes and creates spaces for engagement + information library that are accessible  **[Outcomes]** expand who is in the conversation on the issues that affect us and who has access to it; bridge local and global dialogue and action; make govt accountable to international agreements and commitments | | |
| 2_Learning about Living (LaL) | e-learning system (CD/DVD) based on the Nigerian Family Life and HIV/AIDS Education (FLHE) curriculum.  + **Associated mobile component comprising a Q&A service and online competition** | Nigerian context  Young people  Trained teachers to deliver program  National Commission for Colleges of Education (NCCE)  National Agency for the Control of AIDS (NACA)  Federal Ministry of Education  Civil society organisations  Education as a Vaccine (EVA) in Abuja; Action Health Incorporated (AHI) in Lagos; Girls Power Initiative (GPI) in Calabar  NGOs  OneWorld Uk Buetterfly Works Netherlands  Action Aid Nigeria (AAN)  Funders  Oxfam  Novib Netherlands The John D. and Catherine T. MacArthur Foundation  David and Lucile Packard Foundation Butterfly Works  Global Fund | **[Enabling conditions]** Functional computers and regular electricity; Marketing and promoting the project through mass media is required for better reach, especially of the MYQ&A.  **[Triggered Mechanisms]** make informed decisions; *self-efficacy & agency*  **[Outcomes]** enhance the physical, reproductive and mental well-being; prevent HIV&AIDS, sexual and gender based violence; provision of accurate, non-judgmental information about SRH and gender empowerment   - LaL is a set of educational tool through ICT (I) that can be used in- and out-of-school to enhance the physical, reproductive and mental well-being (O) of young people (A) by providing new routes and access to accurate information (C) to make informed decisions (M) about their sexual health, and to prevent HIV&AIDS, sexual and gender-based violence, and to reduce associated mortality and morbidity (O). - LaL (I) has performed well in achieving its core mandate of using ICT to provide young people (A) with accurate, non-judgmental information about sexual and reproductive health/HIV/AIDS and gender empowerment (O). The electronic version of the FLHE can work given the right environment namely functional computers and regular electricity (C). It has been able to foster creating links (M) with government and civil society and other stakeholders.   **[Enabling conditions]** Mobile services offers anonymity and competition (*game*)  **[Triggered Mechanisms]** *Sense of safety (not judged*); empowering, engaging *(via extrinsic motivation*), *self-determination*  **[Outcomes]** LaL extended beyond schools and community centres – wider reach   - In addition to the e-learning tool, two further services involving mobile phones to further engage (M) young people (A) were launched. These are the Question and Answer services (MyQ & MyA). SMS (I) in particular offers the chance of anonymity (C) for those questions on sexuality that young people may not feel comfortable discussing (M) out loud. The answer service (MyA) is a monthly competition programme which allows young people to engage more (M) with SRH issues. This mobile component (I) has helped extend LaL beyond schools and community centres (O) thus empowering (M) young Nigerians to gain access to sexual and reproductive health information on their own terms (M). | | |

| 3_New media strategies for SRHR campaigns among young people in informal settlements | New media: umbrella term that includes iPods, Netflix, smartphones, video on demand, iTunes, e-mail, video games, YouTube, Facebook, Skype, text messages, Twitter, Instagram, Imgur, LinkedIn, Flickr, Google+, Reddit, among others  Key messaging  School-web-based solutions  Combination strategy  New media-geographic-based strategy  Digital billboards  Sustained solo strategy | Kenya context  AYA  Media managers  Parents  Church | **[Enabling conditions]** Integrating appropriate new media technologies  **[Triggered Mechanisms]** Enlarged virtual communication spaces  **[Outcomes]** Youth engagement, SRHR dialogues with multiple actors   - Young people [A] must be engaged [O] in interactive communication by integrating appropriate new media technologies [C]. By so doing, virtual communication spaces are enlarged [M], which mutually benefit SRHR dialogues [O] among the young people, policymakers, and development agencies [A].   **[Enabling conditions]** Control the message content; timing of a message  **[Triggered Mechanisms]** Effective and strategic communication  **[Outcomes]** Successful new media campaigns   - According to stakeholders [A], messaging is everything in a campaign. Without the message, there is no campaign. Failure to control the message [C] on new media platforms can alter the trajectory of a message [O]. The timing of a message is crucial [C] because wrong timing constitutes a challenge.   **[Disabling conditions]** Limited budget  **[Triggered Mechanisms]** Resource barrier  **[Outcomes]** Reduced campaign launches   - Budgeting is often limited [C], leading to reduced campaign launches [O]. Cultural beliefs or taboo topics [C] hinder a free discourse [O] as far as SRHR is concerned. The lack of political will and unwilling curriculum developers [C] are also obstacles.   **[Disabling conditions]** Lack of follow-up after a campaign; information not moderated correctly; limited online space  **[Triggered Mechanisms]** Neglect of content; information distortion/misinterpretation  **[Outcomes]** Myths, misconceptions, and unprofessional opinions will thrive   - Lack of follow-up after a campaign [C] has been rolled out can affect it [O]. Disseminators [A] may not know whether the message was received and whether the target audience received the same message or whether it was distorted [O]. If the information is not moderated correctly [C], then myths, misconceptions, and unprofessional opinions will thrive [O]. - New media only allows a limited number of words, for example, Twitter (280 characters), forcing message dissemination in threads [C]. Limited space [C] is a breeding ground [M] for misconceptions and misinterpretations[O]. |
| --- | --- | --- | --- |
| 4_SMS text message intervention for AYA Peru | Sending SRH information and questions SMS over mobile phones in Project “ARMADILLO” (Adolescent Reproductive Mobile Access and Delivery Initiative for Love and Life Outcomes) | Peru context  AYA  Peruvian MOH or MINSA  Universidad Peruana Cayetano Heredia (UPCH)  adults in each community (“gatekeepers”) who had worked extensively with adolescents and youth  Ministry of Education (MINEDU)  International  WHO | **Theme: Knowledge as Power (AGENCY)**  **[Enabling conditions]** Privacy, wide availability, dissemination of information  **[Triggered Mechanisms]** Informed decision making,  **[Outcomes]** Improvements to young people’s SRH   - Improvements to young people’s sexual and reproductive health (SRH) outcomes (O) require the dissemination of information (C) about SRH topics to young people to give them the tools and support to make informed decisions (M). Dissemination of information through text messages or SMS (I) is a promising strategy given the privacy of SMS and the wide availability of cell phones (C) among adolescents and youth (A)   **Theme: Engaging/appropriate content**  **[Enabling conditions]** language adaptation to youth, content adaptation to gender, specificity of messages  **[Triggered Mechanisms]** *Ease of understanding*  **[Outcomes]** Increased knowledge and making informed decisions for SHR/R; better able to provide feedback and opinions   - language of the messages (I) was intentionally drafted to be conversational, rather than authoritative (C), as well as simple (at the level of an adolescent (A) and colloquial. Make sure that the messages were very specific (C); When concepts were much better defined (C), as in the SMS, young people (A) were better able to understand (M) and provide opinions and feedback (O).   **Theme: Threats to youth SRHR**  **[Disabling conditions]** conservative culture of Peru, longstanding influence of the Catholic Church, generationally perpetuated violence against women, children and adolescents, reproduced by its cultural acceptance, weak reporting mechanisms, lack of information regarding rights  **[Triggered Mechanisms]** holding onto existing values/beliefs, pressure to conform to old social norms, distrust of providers and institutions  **[Outcomes]** Ministry of Education (MINEDU) recently weakening components of the National Basic Sexuality Education Curriculum that discussed gender norms and gender equality   - The current conservative culture of Peru, where the influence of the Catholic Church is longstanding (C) and influences many people’s beliefs (M) regarding SRH. Pressure (M) from conservative social groups in the country (C) has resulted in the Ministry of Education (MINEDU) recently weakening components of the National Basic Sexuality Education Curriculum that discussed gender norms and gender equality (O) - violence against women, children and adolescents in Peru is perpetuated generationally (C) and is reproduced by its cultural acceptance, weak reporting mechanisms, lack of information regarding rights (C), and the population’s distrust (M) of providers and institutions that should offer protection |
| 5_ “The Times They Are A-Changin”: using technology for ASRHR in the 25 years since ICPD | Technology in general | Adolescents  Global | **[Enabling conditions]** Technology providing scale, connection, privacy  **[Triggered Mechanisms]** *Social influence,* sense of safety/privacy  **[Outcomes]** Individual and group behavioural changes; solution developed in one part of the world can catalyze change in another context   - Technology [I] offers scale and can connect adolescents [A] with information, networks, products and services [C], with privacy and without gatekeeping [M] from community and providers [A]. The content on technology platforms inﬂuences how people act, and how they think and feel [O], connecting them to others who are similar or dissimilar to them. A solution developed in one part of the world can catalyze change in another context [O].   **[Enabling conditions]** Using technology platforms on human rights and social justice issues.  **[Triggered Mechanisms]** Build/strengthen virtual communities; facilitate transfer of skills and tools.  **[Outcomes]** Create and curate public opinions and social discourse; build grassroots constituencies across different settings; political organisation; establish pan-global support.   - Technology [I] has been used by ASRH programmes to create and curate public opinions and social discourse [O]. Advocacy movements have harnessed this well, through social media, chatbots, hotlines, using technology platforms on human rights and social justice issues [C], to build grassroots constituencies across different settings [M]. Movements like SheDecides, MeToo, Abort the Stigma have used social media to build virtual communities and transfer tools [M] for political organisation [O], transcending geographical distances to establish pan-global support [O].   **[Enabling conditions]** Vulnerable, hidden and hard to reach populations, and those experiencing subversive healthcare services, or restrictive policies and communities  **[Triggered Mechanisms]** *Opportunity to access* information and SRH services  **[Outcomes]** provides information and counselling services around abortion and reproductive health that are otherwise illegal   - Technology can expand access to SRH services, reaching even the most marginalised and vulnerable hidden and hard to reach populations, and those experiencing subversive healthcare services, or restrictive policies and communities [C]. Initiatives such as The A Project in Lebanon, an online resource [I] that provides information and counselling services around abortion and reproductive health that are otherwise illegal [O], exempliﬁes how technology can address needs of vulnerable populations [A].   **[Disabling conditions]** Digital spaces can reﬂect social hierarchies and regressive norms, and provide fertile ground for the perpetuation of stratiﬁcations, targeted harm and discrimination, through invasions of privacy and threats to cybersecurity.  **[Triggered Mechanisms]** Shape and perpetuate regressive norms.  **[Outcomes]** Negative impact on human rights.   - Technology can transform social and gender norms but is also shaped by them [M]. Digital spaces can reﬂect social hierarchies and regressive norms, and provide fertile ground for the perpetuation of stratiﬁcations, targeted harm and discrimination, through invasions of privacy and threats to cybersecurity [C]. The advent of new technology [I] has ushered in novel security threats and responses to these threats, include states’ exercise of power to surveil their populations, often criticised for the negative impact on human rights [O].   **[Disabling conditions]** Disadvantaged identity, accessibility, ability, literacy and privilege  **[Triggered Mechanisms]** *Position of disadvantage, loss of voice and agency*  **[Outcomes]** Deﬁne and determine an individual’s experiences; societal restrictions to access.   - Identity, accessibility, ability, literacy and privilege [C] can deﬁne and determine an individual’s experiences [O] online, and disadvantages along these spectra could get transferred and further exacerbated in the digital realm. Vulnerable groups, including adolescents and younger people [A] may also be vulnerable online, and this can lead to societal restrictions on their access [M] and use of the same technology that could bring great beneﬁts to them.   **[Disabling conditions]** Developers of technology-based solutions are not those who advocate for ASRH issues  **[Triggered Mechanisms]** *Ignorance to ASRH* *issues; less able to sift out subtle/nuanced online discrimination*  **[Outcomes]** Replication of offline discrimination   - Developers of technology-based solutions are often not those who advocate for ASRH issues [C], so dialogue on design can prevent the replication of the discrimination that exists ofﬂine [O].   **[Disabling conditions]** Technology enables real-time data capture and analytics.  **[Triggered Mechanisms]** Data trail that can be used for analysis; complicated informed consent process; possible surveillance tool  **[Outcomes]** Replication of offline discrimination; compromised AYA privacy, SRH & wellbeing   - While technology [I] has enabled real-time data capture and analytics [C], this has also raised ethical concerns [O]. Young people [A] seek information on SRH digitally with hope that their identity and searches remain concealed. However, technology allows a data trail that can be used for analysis [M]. Consent to use such data, assurances of privacy and not causing harm are not straightforward [M]. Despite merging of data and protection, information can be intrusive and implications can result even in localised settings. In highly restrictive contexts, surveillance [M] of the online behaviour of young people can have a backlash on their safety and wellbeing [O]. |
| 6_ “Stay strong! keep ya head up! move on! it gets better!!!!”: resilience processes in the **healthMpowerment** online intervention of young black gay, bisexual and other men who have sex with men. | HealthMpowerment.org (HMP) is an internet and mobile phone-based intervention.  Anonymous online space  Online forums (for sharing life experiences, health experiences, and react to conversations about research with YB GBMSM | Young Black GBMSM regardless of HIV status | **[Enabling conditions]** peer-level support online; affiliation.  **[Triggered Mechanisms]** sense of connection; self-affirmation  **[Outcomes]** Foster resilience, reject judgmental or stigmatizing views   - peer-level support [C] fostering resilience [O] - importance of aﬃliation [C] and ﬁnding similar others [C] for self-aﬃrmation [M]. - As participants [A] were able to connect with [M] similar others [A] through HMP [I], they shared self-aﬃrmation and encouraged each other [M] in rejecting judgmental or stigmatizing views [O].   **[Enabling conditions]** tone and anonymity  **[Triggered Mechanisms]** facilitated expression *(voice)*  **[Outcomes]** where they could discuss sex and sexuality candidly and without judgement   - The tone and anonymity [C] oﬀered by HMP [I] might have facilitated the expression of sex-positive norms and generated more candid questions and discussions than might have been provided in-person. This aspect of resilience seemed particularly important for the young Black GBMSM [A] in this study who had limited access to people or spaces where they could discuss sex and sexuality candidly and without judgement [O].   **[Enabling conditions]** create spaces where communities can connect and support one another; include program objectives that foster exchanging social support.  **[Triggered Mechanisms]** resilience processes  **[Outcomes]** where they could discuss sex and sexuality candidly and without judgement   - Interventions that create spaces where communities can connect and support one another [C] could leverage resilience processes [M] by intentionally including program objectives that foster exchanging social support [C], engaging in health promoting cognitive processes, enacting healthy behavioral practices, and empowering others. |
| 7_ HealthMpowerment.org: Building Community Through a Mobile-Optimized, Online Health Promotion Intervention | HealthMpowerment.org (HMP) is a mobile phone-opti-  mized, online intervention | YBMSM and TW aged 18 to 30 | **[Enabling conditions]** safe space, facilitated peer support  **[Triggered Mechanisms]** sense of connection; self-affirmation  **[Outcomes]** explore opinions, feelings, and prejudices in dialogue with their peers (voice)   - HMP [I] was designed specifically as a safe space [C] in which HIV-positive and HIV-negative YBMSM/ TW [A] can explore their opinions, feelings, and perhaps even prejudices in dialogue with their peers [O]. HMP’s social networking features allow users to provide and receive emotional support [C], which may enhance understanding and feelings of belonging [M] among YBMSM/TW [A]. - HMP was designed to appeal to those YBMSM/TW who are looking for tailored messages and information as well as those who had been reluctant or never afforded the opportunity to communicate with peers or health care providers. |
| 8_mHealth | mHealth platform was designed as an interactive mobile phone quiz game in which participants could win airtime (i.e. mobile phone credit that can be used for making calls or sending texts) for texting correct answers to SRH questions | Female senior high school students aged 14-24  Ghana – Greater Accra region | **[Enabling conditions]** Rapid expansion of mobile phone ownership in LMICs  **[Triggered Mechanisms]** cost-effectiveness in reach  **[Outcomes]** increase access to health information, knowledge and outcomes, engage AYA with (low SRH knowledge, adolescents with early sexual debut, and adolescents with low parental support)   - With rapid expansion of mobile phone ownership in low- and middle-income countries (LMICs) over the past decade [C], the field of mobile health (mHealth) [I] has emerged as a novel and potentially cost-effective way to increase access to health information and improve health knowledge as well as health outcomes [O]. - mHealth programs are not only an effective tool to increase SRH knowledge overall in the studied setting, but that these programs can also engage and increase SRH knowledge of adolescents from key target populations who are at higher risk [A] of poor SRH outcomes, including adolescents with low parental education, adolescents with low SRH knowledge, adolescents with early sexual debut, and adolescents with low parental support [C].   **[Enabling conditions]** Active engagement in using mHealth  **[Triggered Mechanisms]** Motivation to learn  **[Outcomes]** greater knowledge of reproductive health in both the short-term and longer-term   - Program engagement [C] also appears to be important for knowledge absorption [O]. We found that those who interacted more with the program had significantly greater knowledge of reproductive health in both the short-term and longer-term [O], compared to those who did not interact. Those who did not reply to messages still received the reproductive health information at the end of each week; however, knowledge improvements were minimal at 3 months and not significantly different from the control at 15 months. We cannot interpret the differences causally, as there may be confounding factors such as motivation [M] that affect both desire to interact with the program and knowledge. |
| 9_CrowdOutAids | CrowdOutAIDS (participatory online project)  Social media  Crowdsourcing | Young people online age 15-29  UN CPD  UNAIDS  Youth organisations | **Theme: Involvement of primary stakeholders (AGENCY)**  **[Enabling conditions]** youth participation, accelerated new ICTs, distribution and broadcasting information and communication  **[Triggered Mechanisms]** sense of ownership, enablement, sense of control, *far-reaching capability, power of open-sourcing/democratic space*  **[Outcomes]** exercise citizenship, *participate in democratic processes,* innovation in public participation, youth to formulate problems and generate solutions   - Participation (C) ensures a greater sense of ownership (M) of proposed solutions, enables (M) young people (A) to exercise their citizenship (O), promotes empowerment (M), and fosters a greater sense of control (M) over their lives and futures. - In order to seize the growing momentum for social change led by youth movements around the world (C), accelerated by new communication technology (C), the UNAIDS Secretariat (A) launched a participatory online policy project called CrowdOutAIDS (I). It used social media and crowdsourcing (I), an innovative approach to public participation (O), to enable (M) young people (A) to both formulate the problems as well as generate solutions (O) to youth leadership and participation in the AIDS response. - Crowdsourcing (I) is a distributed (M) problem-solving and production model. Problems are broadcast (C) to an unknown group of solvers (A) in the form of an open call (M) for solutions. Users - also known as the crowd - submit solutions (O)   **Theme: Identity and representation (VOICE)**  **[Enabling conditions]** Reduced state control of information dissemination  **[Triggered Mechanisms]** Active engagement of youth with online content, increasingly diverse voices  **[Outcomes]** Influences how young people access online SRHR information, and what content gets created and shared online.   - Introduction of social media (I) has changed the relationship between source of message and the audience (O), where the audience no longer passively consumes media content, but actively engages in creating it (M). This marks a shift from a one-to-many to a many-to-many model of communication online, which has implications for how young people access information about sex and sexuality as well as the type of information available (O). Ownership has moved from government-controlled public health agencies with state-sanctioned messages to multiple and diverse voices in the online environment (C), where the audience can now engage, reinterpret and generate sexual health information (O).   **Theme: Involvement of primary stakeholders (VOICE)**  **[Enabling conditions]** *Infrastructure for ICT*  **[Triggered Mechanisms]** enable direct public participation, *democratic participation*  **[Outcomes]** Shape priorities for public policy and planning, legal environment; increase transparency of and trust in public institutions and processes   - Potential of social media (I) within the field of public health is not confined to providing health information and promotion. This new technology could also be used to enable direct public participation (M) in shaping priorities for public policy and planning (O), as well as shaping the legal environment (O), to increase transparency of and trust in public institutions and processes (O).   **Theme: Progressive sense of place as resource (AGENCY)**  **[Enabling conditions]** Creating virtual space/platform  **[Triggered Mechanisms]** Connecting young people, space for sharing and synthesizing knowledge, enabling problem solving, collective action/*power*  **[Outcomes]** Co-production of policies, establish network of youth activists and organisations   - The "virtual" participatory policy process (I) was implemented via the online platform CrowdOutAIDS.org (I), and was conceptualized around four simple objectives: connecting (M) a community of interested young people (A); creating a space (C) where young people could share (M) their experiences, ideas and information; synthesizing this crowd's knowledge (M) and enabling them to find solutions (M); and lastly, collective action (M) through co-authoring the final strategy document (O), and the establishment of a network (O) of youth activists and organizations (A) that the UNAIDS Secretariat (A)could work with to implement the strategy.   **Theme: Progressive sense of place as resource (AGENCY)**  **[Enabling conditions]** Will among young people for changing the taboo surrounding talk of sex  **[Triggered Mechanisms]** Sense of openness to discuss SRH and free to break the silence from taboos (*safety/confidence*)  **[Outcomes]** *Changing the public discourse of SRHR with young people to be more open and discusive.*   - Across regions, whether offline or online, participants agreed on the need to be open, free and break the silence (M) around talk of sex and relationships. "Openness to me refers to that state where you are free to talk about sex or relationships in public places (M) or to anyone with whom you like. It means a kind of freedom (M) to me where no one has to fear (M) for what they speak or with whom they are comfy”   **[Enabling conditions]** Using hybrid social media tools and recruiting volunteers from the online forums to host offline discussions  **[Triggered Mechanisms]** Minimized bias, and the inclusion of young people from across different educational, socioeconomic, religious and political orientations  **[Outcomes]** creation of a unique space for gathering diverse perspectives, where all were accorded value   - Through the use of a hybrid (C) of social media tools (I) and recruiting volunteers from the online forums to host offline discussions (C), we sought to minimize bias and include (M) young people from across different educational, socioeconomic, religious and political orientations. The end result was the creation of a unique space for gathering diverse perspectives, where all were accorded value (O).   **[Enabling conditions]** Provision of (safe/open) space  **[Triggered Mechanisms]** Engage in constructive discussions; collective action  **[Outcomes]** SRHR action and influencing policy   - CrowdOutAIDS (I) process demonstrated that when youth are given the space (C), they can constructively discuss their differing perspectives (M) and work together to create recommendations (M) for action and a policy document that puts forward what they collectively identify as their priority issues (O).   **[Enabling conditions]**  **[Triggered Mechanisms]** direct communication channel; horizontal engagement; generated ownership, increase commitment of youth, integrate grassroots perspectives  **[Outcomes]** strategy development, policy document, young people’s engagement and participation in policy strategy and processes; empowerment and mobilization.   - CrowdOutAIDS (I) process enabled thousands of young people (A) to directly engage (M) with the UNAIDS Secretariat (A) on issues of HIV and sexuality, and priority and strategy development (O). Young people were given the opportunity to engage in an open and horizontal process (M), debating key issues (O). - The crowdsourcing approach (I) generated ownership (M) and commitment (M) of young people (A) to the final outcome document (O) and showed that despite the digital divide, online tools (I) can effectively be used to mobilize for offline action (O). Through leveraging social media and crowdsourcing, it is possible to integrate grassroots perspectives (M) from a wide range of young people across the globe into high-level strategy and policy processes (O). This new model of engagement and participation should be further explored for community empowerment and mobilization (O).   **Theme: Threats to youth SRHR**  **[Disabling conditions]** *local presence of cultural taboos around sex (heightened by online public discourse?)*  **[Triggered Mechanisms]** Exacerbated fear of being judged (online/offline); *public backlash*  **[Outcomes]** *Decreased will of changing existing taboos, no behavioural/attitudinal change of public towards SRHR in youth; towards more regressive SRHR policies*   - Respondents noted the will among young people for changing the taboo surrounding talk of sex. This was often accompanied by a fear of being judged (M). "I would like to change the taboo around sex (C), which is wrong. Before, we did not talk about sex because it was sacred. Now, everything is trivialized, but we still do not really talk about it. We don’t know what we are afraid of, even though the facts are alarming: STIs and deaths from AIDS, pregnancy, abortion (O). |
| 10_ Coach Tumi: Pretesting an mHealth Intervention for At-Risk Adolescent Girls in Soweto, South Africa: Studying the Additive Effects of SMSs on Improving SRHR & Outcomes | Mhealth  2-way SMS campaign *(Coach Tumi)* delivered through a single-sex, soccer-based HIV prevention programme.  Unstructured Supplementary Service Data (USSD) line using Open Data Kit (ODK). A USSD line is a menu of options that facilitates a conversation about a service between a person and a phone and can be provided at no cost for the user. | At-Risk Adolescent Girls 11-14  Soweto, South Africa  Coaches | **[Enabling conditions]** Connecting with SMSs on a weekly basis  **[Triggered Mechanisms]** *reinforcement, exposure* ?  **[Outcomes]** increase participants’ uptake of sexual reproductive health and rights services in their communities; and reach participants regardless of whether or not they own mobile phones.   - [I] keeps participants engaged with important discussions during and after the intervention by connecting with SMSs on a weekly basis [C]; increase participants’ uptake of sexual reproductive health and rights services in their communities; and, reach participants regardless of whether or not they own mobile phones. [O]   **[Enabling conditions]** anonymous text service, safe space, age-appropriate content.  **[Triggered Mechanisms]** *sense of security and privacy, relevance*  **[Outcomes]** engage youth with knowledge and information about local sexual reproductive health and rights services.   - FGD participants[A] believed that the Coach Tumi prototype [I] could provide a promising way to engage youth with knowledge and information about local sexual reproductive health and rights services [O] in a private environment [C]. - According to coaches, the USSD model offers girls a safe space [C] to ask private questions about their sexual reproductive health and rights outside of SKILLZ Street: Additionally, coaches [A]felt that the topics presented in the main menu are consistent with key messages within the SKILLZ Street curriculum and are appropriate for girls age 11-14 [C]/[A]. |

| 11_Tune Me | Tuneme.org  TuneMe – a mobile site (mobisite) designed for low- and high-end devices in environments where high data charges and poor network coverage combine to limit access to online services | UNFPA East and Southern Africa Regional Office  Praekelt Foundation, Ford Foundation  DFID  Young people  Zambia, Malawi, Zimbabwe, Namibia, Botswana, Swaziland and Lesotho | **[Enabling conditions]** social features and content designed to engage users  **[Triggered Mechanisms]** motivation  **[Outcomes]** make informed choices   - Through social features and content designed to engage users [C] rather than lecture them, Tune Me [I] aims to equip adolescents [A] with the information and motivation [M] they need to make informed choices [O].   **[Disabling conditions] In** the socio-cultural contexts of the countries, where intergenerational dialogue about sexual and reproductive health is still considered sensitive, and young people often report that they face discrimination by health workers  **[Triggered Mechanisms]** private and confidential opportunity without facing stigma and discrimination 🡪 *sense of safety and comfort 🡪 agency*  **[Outcomes]** overcome barriers that young people experience in their search for SRH information   - use of mobile phones [I] as a method for reaching out to young people with SRH information is effective as it can overcome barriers that young people experience in their search for SRH information [O]. For example, in the socio-cultural contexts of the countries, public communication, especially intergenerational dialogue about sexual and reproductive health is still considered sensitive, and young people often report that they face discrimination by health workers [A]when they approach the clinics for SRH information and services [M]. Accessing SRH information through their phones [I] therefore provides a welcome private and confidential opportunity without facing stigma and discrimination [M], and the option to access the information at a place and time convenient to the user [A].   **[Enabling conditions]** TuneMe’s newest feature finds youth-friendly clinics by geo-location, including feedback mechanisms to track quality of care  **[Triggered Mechanisms]** the ‘go to’ mobisite for reliable, realistic, and holistic sexual and reproductive health information in the region  **[Outcomes]** Obtain accurate sources of information to shape SRH   - Aside from SRHR content in the form of articles, stories, and testimonials, TuneMe’s [I] newest feature finds youthfriendly clinics by geo-location, including feedback mechanisms to track quality of care [C]. TuneMe is well underway to become the ‘go to’ mobisite for reliable, realistic, and holistic [M] sexual and reproductive health information in the region. This is particularly important given the many competing sources of information, of various quality, that young people are exposed to each and every day that potentially shape their sexual and reproductive health [O]   **[Enabling conditions]** local young people and experts review the content so that certain aspects are adapted and translated for local audiences;  **[Triggered Mechanisms]** relevance to needs and motivation  **[Outcomes]** higher user engagement   - For each country in which TuneMe [I] is running, local young people and experts review the content so that certain aspects are adapted and translated for local audiences [C] to ensure relevance [M] and improve engagement [O] while being presented in a youth friendly edutainment style that respond to the expressed needs of young people.   **[Disabling conditions]** countries with harmful traditional practices e.g. early marriage  **[Triggered Mechanisms]** increase knowledge, agency  **[Outcomes]** Empowerment   - One concrete example would be content around harmful traditional practices [C] where countries such as Zambia and Malawi would discuss early marriages and its consequences, particularly for young women [A]. TuneMe further presents the opportunity to inform its users of the local sexual and reproductive health related laws and policies [M] such as national age of consent to sex, marriage, access to services such as HIV testing or contraception. - TuneMe was built with its end users in mind. While mobile phone (including low-end smartphone) penetration rates continue to grow rapidly in the Sub-Saharan Africa region, the site itself is optimised for use on any device – including low-end devices with limited functionality. TuneMe supports any device that uses a browser – both WAP (Wireless Application Protocol) and HTML. TuneMe is supported by Mobile Network Operators (MNOs) on Free Basics. Free Basics is a partnership between Facebook, Praekelt Foundation and local MNOs to provide free access to content via the MNO’s operator deck.   **Enabling conditions]** allow local youth groups to take ownership of the content [C]  **[Triggered Mechanisms]** Empowerment  **[Outcomes]** learn to run its mobisite and Facebook platform, and use them as advocacy tools in campaign activities.   - TuneMe’s [I] long-term goal, besides behavioural change of young people [A], is to empower [M] local youth groups [A] to take ownership of the content, learn to run its mobisite and Facebook platform, and use them as advocacy tools in campaign activities [O].   **[Disabling conditions]** environments where mobile phone usage is high but data and Wi-Fi infrastructure is limited; lower-income areas  **[Triggered Mechanisms]** TuneMe is adaptable and accessible to a wider range of technology  **[Outcomes]** Increase access to SRHR resources on TuneMe   - This innovation fits well in environments where mobile phone usage is high but data and Wi-Fi infrastructure is limited [C]. TuneMe’s [I] strength is in its ability to be adaptable and accessible [M] to a wider range of technology used by young people [A], particularly in lower income areas [C]. |
| --- | --- | --- | --- |
| 12_#ByeTaboo | Argentina national  SRH online platform #ChauTabú (#ByeTaboo)  For creation of virtual online communities and spaces for SRH education. | Target group  Young Argentinians (15-29yo)  Political/institutional actors  Government of the City of Buenos Aires  Youth General Direction, a department of the Deputy Chief of Government of the City of Buenos Aires  Public Health Communications team  Civil society  Key civil society organisations  Leading national HIV/AIDS, LGBT and women’s rights civil society organisations  Church and Christian religious associations (opposition)  Parents  Health education professionals  Editorials in the leading national newspapers (detractors) | **Theme: Progressive sense of place as resource**  **[Enabling conditions]** connected, private, alternative, non-exclusive, rights-based non-heteronormative approach  **[Triggered Mechanisms]** autonomy, enablement, direct communication channel **[Outcomes]** interaction, expression, networks of social relations, understanding   - When SRHR is advocated via online platform #ByeTaboo (I), its digital nature and its treatment of sex and sexuality through a rights-based non-heteronormative approach (C) provides young people (A) a ‘place’, where networks of social relations (O) and understandings occur(M). This allows a sense of place which is extroverted (C) - creating links with the wider world, which integrates in a positive way the global and the local (O).      - Underlying the development of #ChauTabú (I) is the insight that different ‘places’ are necessary for the provision of SRHR information (C), as these enable (M) users (A) to interact and express themselves (O) with differing degrees of freedom (M). - A ‘progressive sense of place’ (C), in which taboos are addressed and cultural norms challenged (O), was followed in the creation of #ChauTabú (I) as a secure, discreet online space (C). This space would provide information and also work as a channel of direct communication (M) between the government and the youth (A). Thus, this becomes an alternative and non-exclusive space (C) for a rights-based SRH approach, which can provide greater privacy (M) for young users (A) than ‘traditional’ spaces. - Argentina (A) has a track record of some progressive policies with regard to sexual rights (C), which may have helped public trust (M) in the service (I).   **[Enabling conditions]** legal frameworks already in place, positive framing of sex and sexuality, stressing the importance of individual choice and positive rights based on informed decisions and responsible practices  **[Triggered Mechanisms]** empowerment, advocacy  **[Outcomes]** make informed SRHR decisions, sociocultural, political and legal innovations that can advance SRHR   - The site (I) is not limited to SRH and emphasises youth rights (O). One of the main focuses across #ChauTabú is young people’s (A) rights regarding access to SRH services and information. This refers both to *legal frameworks already in place, and to framing sex and sexuality as pleasure, stressing the importance of individual choice and positive rights based on informed decisions and responsible practices* (C) . Underlying this provision of information is the argument that sex education empowers people (M), allowing them to make informed decisions (M) about their lives and bodies, whereas a lack of information impoverishes and disempowers people (Cornwall and Jolly 2006; Armas 2006) and may lead to unplanned and/or undesired outcomes. - #ChauTabú (I) and other similar initiatives should be conceived as having the potential to go beyond their immediate value as information resources and rather be seen as platforms (C) from which to advocate (M) for sociocultural, political and legal innovations that can advance sexual and reproductive rights (O)   [***Disabling* conditions]** Sociocultural differences in other countries, societal progressiveness  **[Triggered Mechanisms]** *willingness to accept*  [**Outcomes]** potential for social controversy   - If #ChauTabú’s (I) main features – namely imagery, language, a rights‑based approach and a fluid understanding of sex, sexuality and gender (C) – were to be adopted and adapted in other countries (A), there is potential for social controversy (O). *M = willingness to accept due to sociocultural differences (C)?*   **Theme: Knowledge as Power (AGENCY)**  [**Enabling conditions]** low cost, far reach, programming flexibility, customizable information  **[Triggered Mechanisms]** self-directed learning, instil agency, self-determination **[Outcomes]** challenge power relations, address taboos, SRHR information gains universal value   - The low cost, potential reach to remote and/or underserved populations, programming flexibility and customizability of information (C) makes technology-based interventions – including online platforms (I) work when SRHR information available becomes of universal value(M). Furthermore, the platform is interactive, private and autonomy (C), which allows for young person’s (A) (drive for) self-directed learning (M). - #ChauTabú is to a sexual education tool (I) that addresses taboos (O), by providing information not only about sex and sexuality, but also about young people’s rights (C) in order to challenge power relations and instil agency (M) for YP (A) to determine their needs. - using audiovisual resources (e.g. illustrations, animations) (C) the initiative (I) could help overcome illiteracy barriers (M), and it has potential to debunk taboos or misconceptions (O)   **[*Disabling* conditions]** Digital platform used without complementary forms of support and services  **[Triggered Mechanisms]** Reduced opportunity for critical thinking and reflection process  **[Outcomes]** Reduced problem solving ability for SRHR issues   - (-ve) Digital platforms (I) have reduced opportunities for young people’s (A) critical thinking and reflection processes (M), thus undermining their ability to strategise and come up with solutions when faced with SRHR issues (O). This suggests that digital platforms have to be delivered in conjunction with other forms of support and services in order to result in behaviour change (C).   **Theme: Identity and representation (VOICE)**  **[Enabling conditions]** Use of youth/sexual minority images/ language/ aesthetic style, avoid jargons  **[Triggered Mechanisms]** motivation to use, identification, validation  **[Outcomes]** inclusion/respect diversity, provide relevant information for all needs   - The language of #ChauTabú focuses on being biomedically accurate and informative, while using everyday language, including the slang (C) that young people (A) use when talking to their peers. Throughout the site, the use of SRHR jargon or technical/scientific terms was avoided (C). A set of illustrations was commissioned to help develop a user-friendly interface (O). These were intended to be attractive (M) to youth (A), and to offer an identity that users could identify with (M). - The images are inclusive of lesbian, gay, bisexual, trans, queer, intersex (LGBTQI) couples, as well as representing different aesthetic styles (C) adopted by the youth of Buenos Aires (A). The rationale for this imagery was to capture the diversity (O) of the city, and to make users feel represented (M) when navigating the site. - #ChauTabú was innovative in Argentina for being a state-run SRHR platform that comprehensively adopted a non-heteronormative rights-based approach to SRH (C) directed at youth (A). The aim of both the written and graphic content is to adopt an inclusive perspective (M) of sex and reproduction, which considers sexuality and gender as fluid (O). The information provided is relevant for the SRHR of LGBTQI partners, and provides information relevant to trans youth under Q&A (O).   **Theme: Involvement of primary stakeholders (AGENCY)**  **[Enabling conditions]** Support, participation and co-creation with stakeholders  **[Triggered Mechanisms]** *inclusiveness, agency*  **[Outcomes]** Contributing and providing feedback, shared decision-making   - […] support and participation (C) of several key civil society organisations (A). The website content (I) was co-created (C) with leading national HIV/AIDS, LGBT and women’s rights civil society organisations (A), which revised a first proposal, provided feedback for its improvement and approved the final version (O) |
| 13_MyPEEPS Mobile | MyPEEPS Mobile is a mobile, responsive Website that is viewable on small screens and usable with touch screens.  Provides educational information about STIs and HIV for YMSM, builds skills for condom use, and raises awareness of minority stress. | Young MSM (19-25) | **[Enabling conditions]** [I] is informative and useful; easy to use; Important others also using and sharing app  **[Triggered Mechanisms]** Perceived usefulness [I]’s performance of; reduced effort expectancy; social influence  **[Outcomes]** Increased knowledge; higher usage frequency  **Performance expectancy**  Perceived usefulness [M] of MyPEEPS [I[ was ascertained from end users’ [A] summative experiences and perceived knowledge attained [O] while using the app. Most participants noted that the app was informative and useful [C] beyond standard sexual health education. Participants expressed a foreseeable usefulness of MyPEEPS for YMSM who live in different geographic regions (ie, outside of urban settings) [A]  **Effort expectancy** [M] is defined as the degree of ease [C] associated with the use of MyPEEPS [I], including ease of use and complexity. Most end users [A] described the mobile app as easy to use [O].  **Social influence** was applied in this study as the degree to which an individual perceives that important others believe that they would benefit from the use of MyPEEPS app.  Conversely, other participants noted that they would not share this app or content with their partner(s) or friend(s) because of embarrassment, implied stigma, or discrimination:  UTAUT model (Unified Theory of Acceptance and Use of Technology):   1. Performance expectancy is defined as the degree to which an individual **believes** that using the technology will help them attain gains in the outcome of interest 2. Effort expectancy is the degree of ease associated with use of the technology. 3. Social influence is the extent to which an individual perceives that important others believe they should use the technology. These constructs are theorized to drive behavioral intention. |
| 14_My space, my body, my sexual subjectivity: social media, sexual practice and parental control among teenage girls in urban Chiang Mai | Social media | Chiang Mai, Thailand  Young that women, teenagers  Parents | **[Disabling conditions]** Social norms; structural dominant forces  **[Triggered Mechanisms]** Adaptation, resistance against hegemonic powers,  **[Outcomes]** create space for shaping and expressing their sexuality   - while young Thai women can never entirely escape the dominant forces of the state inflicted upon them through school [C], the media and other channels, they are able to create space for shaping and expressing their sexuality [O], through the use of the social media [I]. The ways in which teenage girls use the social media are therefore perhaps best understood as a site of struggle against the hegemonic power [M] imposed by their families, schools and other social institutions. - processes of adaptation and resistance [M]   **[Enabling conditions]** The social media’s role in helping young women to negotiate and restructure ideas, norms and values related to gender and sexuality subjectivity (in contexts of parental control and values of society)  **[Triggered Mechanisms]** making sense of cultural cues and negotiating public life; negotiate sexual subjectivities  **[Outcomes]** sexual autonomy   - The social media [I] play a significant role in helping young women to negotiate and restructure ideas, norms and values related to gender and sexuality subjectivity [C]. Through smart phones and online social network applications such as Camfrog [I], young women in Thailand can display their bodies and express their sexuality within contesting discourses on feminine sexuality that are shaped by the state, consumer values and the media, as well as by young women’s own discourses in a strongly patriarchal society [C]. - according to a study of the utilisation of online social networks by US youth, as well as providing teenagers with a space to work on their identity and status, online social networks also assisted in making sense of cultural cues and negotiating public life [M]. In the present study it was found that social media effectively paved the way for the northern Thai girls to negotiate their sexual subjectivities [M] without the knowledge or interference of their parents. during fieldwork, several teenage girls’ [A] tactics emerged that contested or resisted the mechanisms of parental control [C] described above, enabling them to achieve a certain level of sexual autonomy [O]   **[Enabling conditions]** connect to others; facilitate the establishment of their own secret and public communities; providing an alternative cyberspace  **[Triggered Mechanisms]** assert their agency, adapt, resist  **[Outcomes]** negotiate and challenge traditional forms of femininity   - This study reveals how young women in northern Thai middle-class families [A] creatively use the social media and the Internet. These online spaces allow them to connect to others [C] despite the existence of physical boundaries. Social media also facilitate the establishment of their own secret and public communities [C]. online social networks play an important role by providing an alternative cyberspace [C] for young women (and others) to gather together through online social networks instead of via the conventional physical gathering places. Through social media utilisation [I], young women are able to assert their agency [M] and find ways to negotiate and challenge traditional forms of femininity [O] enforced upon them via the preaching and sermons they receive from school teachers and parents.   Theories  *Very much in line with the concept of ‘power/knowledge’ that Foucault (1990) describes in his History of Sexuality, the Thai state has embarked on a series of processes attempting to control, regulate and suppress young women’s sexuality in northern Thai society.*  *Work of de Certeau (1988), which argues that in contexts where people are constrained by a dominant cultural order, they can adapt it to their own ends.* |
| 15_ Sexual Assertiveness Skills and Sexual Decision-Making in Adolescent Girls: Randomized Controlled Trial of an Online Program | Interactive, Web-based sexual health  program (Health Education and Relationship Training [HEART] | SE United states  10^th^ grade girls (15-16yo)  Rural, low-income high schools | **[Enabling conditions]** Interactive  **[Triggered Mechanisms]** Engagement  **[Outcomes]** increased ﬁdelity to program content; reduced costs; and the potential for broad dissemination   - There are many beneﬁts of utilizing interactive digital technologies [I] to engage [C] youths [A] with prevention messages. These include increased ﬁdelity to program content, reduced costs, and the potential for broad dissemination [O].   **Sustainability of outcomes**  **[Disabling conditions]** Sexual assertiveness and negotiation skills are difﬁcult skills to learn  **[Triggered Mechanisms]** *lack of skills mastery, lack of motivation to sustain healthy SRH behaviours*  **[Outcomes]** Diminished outcomes after certain time period   - Several of these treatment effects had diminished within 4 months—a common ﬁnding among sexual health interventions for youths [O]. This suggests that added material or modiﬁcations are necessary to enhance the potency of the HEART program. Sexual assertiveness and negotiation skills are difﬁcult skills to learn and master [C]—even for adults; thus, youths [A] will likely need ongoing modeling and opportunities to practice and reﬁne these skills. |

| 16_ Sexual and Reproductive Health Rights and Information and Communications Technologies: A Policy Review and Case Study from South Africa | ICT policies in SRHR | South Africa  Focus on marginalised groups (women, girls, peri-rural) | **[Enabling conditions]** ICT policies are aligned with international standards and with South Africa’s international commitments  **[Triggered Mechanisms]** *obligations to fulfil commitments*  **[Outcomes]** The strategy adopts internationally recognised health informatics standards and closely follows the WHO’s approach to e-health, e.g. **monitoring of public health.**   - These ICT policies are aligned with international standards and with South Africa’s international commitments [C]. The strategy adopts internationally recognised health informatics standards and closely follows the WHO’s approach to e-health, namely using ICT for patient treatment, health research, education, tracking of diseases and **monitoring of public health.**   **[Disabling conditions] *policy-related***   - severe dearth of recognition of women’s and girls’ rights in relation to dignity, health, and SRHR - policy vacuum and lack of intersection between ICT and SRHR - Despite progressive policy supporting a wide-ranging set of SRHR issues, the focus has been on MCH while avoiding the more politicised aspects - Disconnect in government departments - Lack of budget for ICT policies   **[Triggered Mechanisms]** *Lack of political leverages for transformations*  **[Outcomes**] Women and girls placed under category of exclusion with no corresponding actions for redress; Women’s and girls’ SRHR are neither respected nor promoted; Communication technologies not seen as a mechanism to enhance women’s and girls’ empowerment; ‘very little interface’ between SRHR and ICT and between the NDOH and DSD   - This research asked, in relation to SRHR and ICT, does it make a difference whether women and girls [A] live in urban, peri-urban or rural contexts?[C] It learned that place is indeed a factor in shaping women’s and adolescent girls’ experiences [M], but that few people – be they policymakers, health experts or ICT implementers [A] – are focusing on this. - Despite progressive policy supporting a wide-ranging set of SRHR issues, the focus has been on MCH while avoiding the more politicised aspects [C] such as reproductive justice , health rights and adolescent sexual pleasure. - The research has shown a policy vacuum and lack of intersection between ICT and SRHR [C]. Instead, policy is being shaped through implementation [C]. - There is a severe dearth of recognition of women’s and girls’ rights in relation to dignity, health, and SRHR [C]. Instead women, more than adolescent girls [A], are identified primarily as a category of exclusion with no corresponding actions for redress [O]. Women’s and girls’ SRHR are neither respected nor promoted in these policies [O]. Communication technologies are not seen as a mechanism to enhance women’s and girls’ empowerment [O]. - This disconnect is also reflected in government departments [C]. Representatives from the DSD [A] explained that their focus has been on government commitments to SRHR, in particular to the ready availability of contraception, violence against women and children and substance abuse. In contrast, the Minister of Health [A] prioritises health information systems over SRHR issues. As a consequence, there is ‘very little interface’ between SRHR and ICT and between the NDOH and DSD: ‘the Department of Health doesn’t talk to us’. Similarly, among researchers and implementers [A]: some SRHR experts [A] have limited understanding of the current use, and extent of, ICT in the health domain and some implementers have only partial perspectives on SRHR and are unaware of their contested nature [O]. - ICT policies have NO corresponding budgets [C].   **[Disabling conditions]** when women step outside conventional gendered roles [C] who support feminist or lesbian rights or who advocate broader SRHR  **[Triggered Mechanisms]** Nature of threat to women’s safety blurred between on- and off-line realities  **[Outcomes]** women’s online safety compromised   - Questions about women’s online safety [O] are expanded when they step outside conventional gendered roles [C]. For women who support feminist or lesbian rights or who advocate broader SRHR [C], receives attacks online through swamping, spamming, threats of personal violence, hate speech and images of extreme sexualised violence are common. As one respondent researching issues of sexuality and the internet indicated: safety is an issue as for women who participate in contentious human rights issues [A] as ICT-users can experience blurring between on- and off-line realities [M].   **[Enabling conditions]** ICT (YAL) as politically progressive;  **[Triggered Mechanisms]** challenge social norms  **[Outcomes]** facilitate social change  Case study: YAL   - YAL, in its recognition of young people’s sexuality, its acknowledgement of SRHR, its willingness to address topics such as abortion and to focus on individuals’ choice, is politically progressive [C]. It seeks to discuss and challenge social norms [M]. ICT and mobile phones are seen by YAL’s implementers as facilitating social change [O]. |
| --- | --- | --- | --- |
| 17_  Crowdmapping | Crowdmapping web app “Free to Be” | Targeted users: young women in Delhi, Kampala, Lima, Madrid, and Sydney  Plan International  Local authorities  Data managers  Website moderators | **[Enabling conditions]** platform allowed sharing stories, generating a vast bank of data  **[Triggered Mechanisms]** challenge the perspectives authorities, generating awareness about the levels of harassment and violence  **[Outcomes]** initiate behaviour change   - Across the complex and often conflicting cultures of this diverse set of cities, the platform [I] allowed girls and young women [A] to share stories, generating a vast bank of data [C] that can hold stakeholders to account for their inaction and acceptance of harassment of girls and young women, which has a direct negative effect on their freedom of movement and ability to participate in city life. The data and the stories from the girls and young women challenge the perspectives of the police, public transport operators, and local government authorities, as well as generating awareness about the levels of harassment and violence that go unnoticed [M], in order to initiate behaviour change [O].   **[Enabling conditions]** Anonymity  **[Triggered Mechanisms]** Sense of privacy / safety to share  **[Outcomes]** girls and young women coming forward (online) with experiences of sexual harassment or abuse in their city   - Free to Be [I], is an excellent collector of stories and impressions of a city from those whose voices are not often heard or recorded. All users were anonymous [C] on the Free to Be Map, with no option to submit a name. This was largely to ensure the safety [M] of participants, so there was no chance of them being identified, but the anonymity proved particularly advantageous for girls and young women [A] outlining experiences of sexual harassment or abuse in their city [O].   **[Enabling conditions]** authorities were included in the discussion about the tool design or in the app launch; involvement of young women in the app design; complementary platforms for advocating with those in power  **[Triggered Mechanisms]** those with power paid attention to the experiences of girls and young women (*raising awareness);* empowering women and girls  **[Outcomes]** Space to discuss findings directly with decision makers; towards real change and accountability taken by those in *power (closing accountability loop*)**;** increased usability and high levels of participation.   - At the city level and to varying degrees, Plan International offices included the authorities in the discussion about the tool design or in the launch of Free to Be [C], thereby ensuring that through the whole process those with power paid attention to the experiences [M] of girls and young women [A]. - The involvement of young women [A] in the design of the Free to Be Map was a crucial factor in its usability and high levels of participation. Plan International’s [A] ongoing work with groups of girls and young women also provided a platform to empower [M] them to use the data and make space for them to discuss findings directly with decision makers. Data alone are powerful, but incorporating modes for use and fora for advocating with those in power [C] are essential to providing opportunities for real change [O].   Technical aspects/features  **[Enabling conditions]** Facilitates accurate geographic coordinates; attractive visual interfaces; Users can view and comment on other submissions; real time  **[Triggered Mechanisms]** Reliability of data; motivation to use app; feelings/dynamics of connection  **[Outcomes]** greater levels of public participation; communicating large datasets using easily digestible powerful methods   - Crowdmapping can improve data accuracy [M] through allowing participants to associate submissions with a specific geographic coordinate. - Crowdmapping can also generate greater levels of participation as people are attracted to [M] visually enticing images and interfaces [C], generating greater levels of public participation than text-based surveys [O]. - Users can view and comment on other submissions [C] creates a powerful dynamic where people can share similar stories, deliberate, and feel connected with one another [M]. - Crowdmapping offers powerful communication tools, in real time/ spatial dataset outputs for communicating large datasets using easily digestible powerful methods [O].   **[Disabling conditions]**   - under-resourced or remote settings - whole communities who lack skills in reading and navigating a mapping system, struggle with a lack of WiFi or data connectivity, or have older hardware which is not compatible with the latest or emerging software - level of detail contained on the base map not reflecting actual situation on the ground   **[Triggered Mechanisms]** *Lack of opportunity to be connected/reached/engaged*  **[Outcomes]** Inaccessibility to app/platform; user discontent, low participation, inaccurate responses.   - In under-resourced or remote settings, there may be people and whole communities who lack skills in reading and navigating a mapping system, struggle with a lack of WiFi or data connectivity, or have older hardware which is not compatible with the latest or emerging software [C]. In both Kampala and Delhi, promoting the website via social and traditional media proved ineffective, generating very few responses. Further, the most vulnerable girls and young women in some of the poorest parts of the city [A] simply could not access this platform [O], due to lack of access to the internet, digital literacy, or exposure to the promotion and information about the site on mainstream media. - In some locations the level of detail contained on the base map does not necessarily reflect the situation on the ground [C]. This challenge has the potential to be a cause for discontent, low participation, or inaccurate responses [O].   **[Disabling conditions]** Not being able to control who is dropping pins and leaving comments (anonymity)  **[Triggered Mechanisms]** online trolling behaviour (invisible identities)  **[Outcomes]** girls and young women stopped sharing stories   - Anonymity can be an advantage of crowdmapping, but also has the limitation of not being able to control who is dropping pins and leaving comments [C]. The Free to Be Maps for all five cities were affected by ‘trolls [O]. This action demonstrates a level of determination to stop girls and young women [A]sharing their stories, suggesting the collective action posed a threat to the patriarchal status quo. The open nature of crowdmapping makes it extremely difficult to prevent such action, so structures need to be in place to moderate and respond [countermeasures]. |
| 18_  Empowering Women in India to Influence Maternal Healthcare Quality Through Mobile Phones and Crowdsourcing | Mobile Monitor for Quality of maternal Care (MoM-QC) | Women (maternal group) in rural Jharkhand  Healthcare providers  White Ribbon Alliance for Safe Motherhood India (WRAI)  Centre for Catalyzing Change  Local NGO  Mobile technology  Gram Vaani  Content moderators  Funder  Merck | **[Enabling conditions]** Interactive voice instead of text; connection with Mobile Vaani (free, already has audience base)  **[Triggered Mechanisms]** Bypass literacy issue with rural women; connectivity  **[Outcomes]** powerful tool for two-way communication to both “push” and “pull” data   - A key goal for this project was to transform the mobile phone—a device conventionally used to make and receive calls—into a powerful tool for two-way communication—that is, to both “push” and “pull” data [O]. Women in rural Jharkhand [A]have relatively low literacy rates [C] thus to ensure ease of use, and enable more women to participate [M], developers [A] opted to incorporate interactive voice response (IVR) technology [I] rather than SMS/text messaging. The IVR prototype [I] was linked to Gram Vaani’s Jharkhand Mobile Vaani, a popular free call-in radio service available to all users in the state. Connecting the platform to this service allowed the service to reach a ready-made audience of users [C].   **[Enabling conditions]** Free incoming calls, low-tech functionality.  **[Triggered Mechanisms]** Overcome costs barriers, enable agency and voice.  **[Outcomes]** Increase engagement and in rating services.   - Incoming calls within the country have been free in India since 2003 [C]. To ensure that participants did not have to bear the cost while they engaged with the platform [M], the system was set up for immediate call backs [C]. - The platform’s [I] simple, low-tech functionality [C] leveraged the ease of cell phone use while giving each caller the power to “give voice” [M] and rate the services they received [O].   **[Enabling conditions]** Availability of information and feedback platform.  **[Triggered Mechanisms]** Empowerment  **[Outcomes]** Make better decisions regarding their own and their family’s health, and to demand quality care   - Fifty-one women who had participated in the MoM-QC pilot were later interviewed to understand the influence of the feedback [C] on their decision on seeking care at health facilities. At least 68% (n = 35) of women shared that the information provided through the IVR [I] platform had empowered [M] them to make better decisions regarding their own and their family’s health, and to demand quality care [O]   **[Enabling conditions]** [I] adapted for rural, illiterate, and semiliterate women; borrowing phones from community health workers, neighbors, and friends;  **[Triggered Mechanisms]** activate the feedback loop; problem-solving, adaptability and tapping on social capital  **[Outcomes]** educate women on quality of care and maternal health entitlements, collect their opinions on quality of care, and develop a community quality of care rating for health facilities   - The MoM-QC [I] pilot project and related efforts to use the digital platform to activate the feedback loop [M]. - Interactive voice response [I] is a suitable platform to reach rural, illiterate, and semiliterate women [C], educate them on quality of care and maternal health entitlements, collect their opinions on quality of care, and develop a community quality of care rating for health facilities [O]. - Socioeconomic constraints limit women from having access to a mobile phone with enough minimum balance on their prepaid card to call at their own costs [C]. Despite this and related barriers to reliable mobile phone access, women found a way [M] to participate in a system that values their input, borrowing phones from community health workers, neighbors, and friends [C] in order to make the missed call and receive call back from the IVRS to give their feedback [O]. Their adaptability and willingness to participate [M] despite access difficulties show enormous potential for a sustainable, large-scale effort [O]. - Health care providers [A] and therefore the facilities (both public and private) [A] are interested in receiving [M] women’s quality ratings. Generating and providing realtime data will require additional backend technology refinements, but would allow for continuous quality improvement for providers [O], and more informed decision making for women [O]. Future testing is needed to determine the most efficient and effective means to encourage providers and health officials to improve care through providing a ***continuous feedback loop*** [still lacking outcome].   Additional information about [I] and it’s theory of change:  Gram Vaani (<https://gramvaani.org/?p=3901>)   - *First, the* ***voice-based nature*** *[C]of the medium, and* ***free of cost [C]*** *to access****, allows a wide range of people to utilize it [O]****.* - *Second, Mobile Vaani advocates* ***editorial policies*** ***that enhance the context****,* ***completeness, and credibility of information [C]*** *on the platform. These properties have been validated to* ***improve the understanding [M]*** *of people about messages floated on the platform by* ***making the messages more contextual*** *through contributions by other people, more complete* ***by creating a culture that encourages diversity and debate of viewpoints from different stakeholders*** *[O], and more credible by ensuring factual correctness of information published on the platform [O]. Such* ***a conversational approach*** *[M] to discussing topics helps* ***build a shared understanding*** *[O]among people.* - *Third, Mobile Vaani provides features to* ***publish selective information*** *[C] on the web, social media, forward it to officials, run pledges, coordinate social movements, etc, to* ***draw the attention*** *[M[ of different stakeholders [A] to important issues and bring about action which can encourage more people to* participate [O]. |
| 19_  Access, Services, Knowledge (ASK) – Youth Empowerment Alliance (YEA) | e&m health | Young people (10-24) in (Uganda, Kenya, Indonesia, Pakistan, Ethiopia, Ghana, Senegal)  SRHR Alliance  Rutgers (lead agency) Amref Flying Doctors the Netherlands CHOICE for Youth and Sexuality  dance4life  Simavi  STOP AIDS NOW! International Planned Parenthood Federation (IPPF).  60 partner organizations, which all form local SRHR alliances in their country | **[Enabling conditions]** [I] offers direct communication channel.  **[Triggered Mechanisms]** Connectivity  **[Outcomes]** Improve access to SRHR information for young people, including marginalized group*   - Direct communication [C] tools such as e&m health [I] were useful to improve access [M] to SRHR information for young people [A], including marginalized groups [A]. *At the same time it was noted that an important group of marginalized youth had no access to internet or mobile phones; therefore, public awareness-raising activities, printed materials, radio programmes and working with peer educators were also important ways to reach them.   **[Enabling conditions]** aligning [I] with the local and national government and obtaining their buy-in where possible; [I] accompanied by more traditional forms of communication  **[Triggered Mechanisms]** Broadened reach  **[Outcomes]** Equal access for marginalized groups with no or limited access to the internet and/or mobile phones   - Strategies that were said to be particularly useful included aligning the programme with the local and national government and obtaining their buy-in where possible [C]. Using direct information tools, including e&m health [I], was valued due to their broad reach [M]. Generally they were also considered useful to reach marginalized groups [O]. - That said, it was found they should be accompanied by more traditional forms of communication such as radio and theatre [C], as some marginalized groups, such as young people living in remote rural areas [A], have no or limited access to the internet and/or mobile phones [C]. |
| 20_The power of youth voices: How youth are holding their health systems accountable for family planning and reproductive health. | MobiSAfAIDS  A web and mobile application used by young people to monitor and report on quality issues they experience at local health facilities. | Young people  MobiSAfAIDS pilot was rolled out in six countries: Eswatini, Lesotho, Malawi, South Africa, Zambia, and Zimbabwe.  Local municipalities  Public health facilities  Public health service providers  Civil society organizations  Funder: Swedish International Development Agency | **[Enabling conditions]** Involve AYA throughout project  **[Triggered Mechanisms]** Agency to shape (I)  **[Outcomes]** (I) continuously refined   - Young people [A] have been involved [M] throughout the project, from pitching the idea and seeking buy-in from the government, to training other young people [C] in the pilot sites. Young people shaped [M] the application’s features, and SAfAIDS continues to receive feedback and to refine and change the system based on their inputs [O].   **[Enabling conditions]** Having young volunteers as in-person support  **[Triggered Mechanisms]** Using knowledge and skills of youth; networked relationships  **[Outcomes]** Solving barriers to technical issues; link AYA with other actors   - Each participating clinic [A] used in-person support from the pilot’s young volunteers [C], who would support [M]young people seeking services with logging service issues, particularly when accessing a smartphone or data was a barrier [O], and also to provide a link [M] between the young person seeking services, the facility, the implementing partners, and SAfAIDS [A].   **[Enabling conditions]** Develop and provide youth-friendly zones  **[Triggered Mechanisms]** Sense of ease; self-efficacy  **[Outcomes]** More AYA asking for help in SRHR matters   - A number of facilities have developed youth-friendly [C] corners or zones where young people [A] can feel confident and comfortable [M] asking for help [O].   **Disabling conditions]** Low penetration of mobile phone usage, lack of access to data, and clinics without free Wi-Fi; recorded information while using digital tools.  **[Triggered Mechanisms]** Disconnection from online intiatives; loss of privacy.  **[Outcomes]** Widened digital divide; Reduced youth participation.   - Low penetration of mobile phone usage, lack of access to data, and clinics without free Wi-Fi [C] were all noted as challenges to youth participation [O] in digital social accountability initiatives. While creative solutions are being tested, such as training youth champions [A] in health facilities to provide support and access for other youth to use digital social accountability tools, there is a need to carefully consider how the digital divide may be further widened through the use of such technology in social accountability [O]. Further, at least one interviewee noted the concern among youth that their information would be shared [M] if it was recorded using digital tools [C].   **[Enabling conditions]** Presence of social media platforms; Use familiar digital tools  **[Triggered Mechanisms]** Organized space for action; Leverage on digital skills; power and agency  **[Outcomes]** Youth articulation of a collective identity and objectives; demystification of SA, share information.   - While the challenge of the digital divide is well documented, there are many places around the world where youth are already collectivized through social media platforms [C]. Where this is the case, digital tools can have an organizing function [M] for youth that allows the articulation of a collective identity and objectives [O]. - Harnessing digital tools that are already familiar [C] to youth can help to demystify social accountability [O]; at the same time, these tools act as an engine of social organization by putting the power directly in the hands [M] of youth to collectivize and share information [O]. |
| 21_  Empowering youth: Use of technology in advocacy to affect social change. | Mainstream forms of ICT categorised as:  Social Networking Sites (Applications, online wall or comments group)  Mobile phones (SMS/MMS)  Other Internet-based technologies (RSS/Twitter/Blog/ podcast) | Youth   Global | **[Enabling conditions]** Speed and ease of sharing information; not limited by time and space or method.  **[Triggered Mechanisms]** Real time connectivity (*knowledge as power*), raise awareness and shape attitudes.  **[Outcomes]** implement the necessary action for social change; change perspectives; promote critical dialogue with policy makers; ease of being involved in simple advocacy action   - Advocates using these technologies can share information at a faster pace [C], recruit more people and use a variety of tools to implement the necessary action for social change [O]. - Raise awareness and shape attitudes [M]. SNS and cell phones make it easy [C] for people to engage in conversation to debate and discuss the issues [C]. Through text messages, posts to SNS discussion boards, wall posts, or e-mails to groups people begin to become more informed about the issues, think about them in a new way, and how it affects their environment [O]. - A related, and particularly powerful, advocacy strategy to raise awareness [M] is photovoice. Photovoice is the process of using photographs to depict a community and using that as a catalyst for change [O] by promoting critical dialogue with policy makers [O]. - Success of advocacy efforts requires persuasive communication with decision makers. With technology, advocates are not limited by time and space or method. A phone call can be made or text message sent from anywhere at any time; a message can be posted on the decision maker’s SNS page. These features make it easy for people to be involved in a simple advocacy action [O]   **[Disabling conditions]** Digital divide due to economic and regulatory factors; selective ﬁltering of messages.  **[Triggered Mechanisms]** *inadequate / poor connectivity*  **[Outcomes]** advocacy causes may get lost in the masses, making it more difﬁcult to recruit and maintain supporters [O].   - In the 1990s, concerns were expressed about the digital divide. Internet and cell phone penetration is lower in several other countries, particularly developing countries [C]. Researchers suggest that the global divide is explained in part income, but also by other economic factors such as quality of regulation. - One potential negative aspect of employing technology for advocacy is the possibility for selective ﬁltering of messages [C]. Because a person has continual access to information through Internet news sites, personal and professional blogs, Twitter, SNS, cell phones, podcasts, and so forth, it is possible that advocacy causes may get lost in the masses, making it more difﬁcult to recruit and maintain supporters [O]. |
| 22_  ‘We have the internet in our hands’: Bangladeshi college students’ use of ICTs for health information. | Mobile phones and internet use | College students    Mirzapur, Bangladesh | **[Enabling conditions]** accessibility of information.  **[Triggered Mechanisms]** challenge existing forms of gatekeeping; enabling / empowerment.  **[Outcomes]** bypass medical and other gatekeepers of SRH information.   - ICTs – particularly laptops and mobile phones with internet access [I]– challenge existing forms of gatekeeping [M]. They make health information accessible [C] and have the potential to undermine medical and socio-cultural gatekeeping practices [M] that control college students’ access to information about sexual and reproductive activities. Access to ICTs thus enables [M] college students [A] to bypass medical and other gatekeepers of SRH information [O].   **[Disabling conditions]** navigating the vast amount of information available through digital technology.  **[Triggered Mechanisms]** apomediation  **[Outcomes]** use digital technology to become educated about a particular subject, and then share this knowledge with peers; potential to restructure health systems   - Health experts and other societal gatekeepers will no longer ‘stand between’ users and SRH information. However, users will still have to find ways of navigating the vast amount of information available to them through digital technology [C]. Eysenbach proposes that gatekeepers will be replaced by other types of intermediaries who ‘stand by’, rather than between, users, helping them to identify relevant and accurate information. These new mediators [A], or apomediators, use digital technology to become educated about a particular subject, and then share this knowledge with peers as and when relevant [O]. ICTs [I] thus hold out a promise to eliminate traditional gatekeepers and, through a process of apomediation [M], to give users ‘direct, convenient access to an abundant amount of health information’, enabling them to share health information amongst themselves [M]. In so doing, this may have the potential to restructure health systems [O].   **[Enabling conditions]** use digital technology as a means to bypass intermediaries.  **[Triggered Mechanisms]** choice (agency); older adolescents 🡪 challenge authority, strive for greater autonomy and find peer-to-peer apomediation attractive  **[Outcomes]** reduce the power of intermediaries   - In theory disintermediation and apomediation [C] provide college students with greater choice [M] as to who might offer health information. The tendency to look for new intermediaries, Eysenbach suggests, is particularly pronounced among young people [A] who, ‘strive to become more autonomous and to reduce the power of intermediaries such as their parents, with peers taking on the role of the former intermediaries [O]. Older adolescents [A] seek to challenge authority, strive for greater autonomy and find peer-to-peer apomediation attractive [M]. - Familiarity with technology also influences the extent to which college students use digital technology as a means to bypass intermediaries [C].   Theory:  *The concept of disintermediation imagines a process of unmediated access to information, which enables people to navigate around gatekeepers, retrieving information which is usually controlled by professional interest groups of various kinds. Evidence from Bangladesh, however, points to the* ***importance of social and cultural gatekeeping in a conservative society****, with constraints (stricter for women and unmarried men) on young people’s sexual health information.* |
| 23_  Instrumentalising the digital: findings from a rapid evidence review of development interventions to support adolescents’ engagement with ICTs in low and middle income countries. | ICT and digital media in general | Youth and children in LMICs | Interesting points for Introduction/Background   - *Intersections among several domains of knowledge – ICTs and digital media, and adolescence (both domains heavily led by research in the Global North), and C4D and ICT4D programmatic interventions in LMICs.* - *The communication strategies which accompanied modernisation interventions* *in the field of development from the 1940s onwards were* ***top-down, decontextualised, and technologically determinist.*** *One of the assumptions on which they were premised was that dissemination of information alone can lead to substantial change and, more specifically, that technical innovation can radically transform remote rural communities in developing nations by pulling them towards Western, urbanised modernity, and comparable economic prosperity.* - *In relation to children and young people, expectations regarding the promise of ICTs are often at their highest, building on the hugely appealing* ***rhetoric*** *of the “****digital native****”* - *Many policymakers and practitioners hope that the proliferation of ICTs and digital media in developing countries can expand the participatory potential of communication for development (C4D) programming. But others argue that the rhetoric around ICTs has provided a new and alluring guise for* ***technocentric modernisation thinking in development*** *(Mazzarella 2010) and is becoming ever more entrenched as the notion of a “****digital imaginary.***   Contextual elements:   - *Access and use depend on resolving a host of challenges associated with electricity, connectivity, cost, digital skills, social acceptability, spatial privacy, and adult permission at the community and individual levels, as well as with commercial viability, investment, and government regulation at the level of the state or region.* - *Providing access without attention to pre-existing skills and social contexts can result in exacerbating rather than ameliorating inequality.*   3 orders of digital divide:   - *First-order digital divide (in access to hardware and connectivity as a function of age, gender, and social class)* - *second order digital divide (in digital media appropriation within families and the development of digital skills and literacies)* - *third-order digital divide (in terms of actual practices of use and, thereby, the benefits that might accrue).*   **[Enabling conditions]**  **[Triggered Mechanisms]**  **[Outcomes]**   - A good example of an initiative seeking to empower [M] adolescents [A] to discuss the issues that concern them while informing local governance policies [O] is UNICEF’s mobile-based platform U-Report [I]. This text messaging communication platform [I] was launched in Uganda in 2011 as a local initiative allowing young people [A] to contribute ideas to governance and decision-making processes that impact on their local communities [O]. - Although in some countries the platform is also used by policymakers, crucially for our argument, evidence that young people’s voices expressed on the platform have any impact on or response from policymakers is slim in the evaluations, and children are not included either in evaluation or policy-making   **[Disabling conditions]**   - divergence between what adult stakeholders expect children (particularly those from low-income areas) to do with digital media and with what they actually do. - interventions have tended to focus on the supply side while ignoring dynamics that structure demand and use   **[Triggered Mechanisms]** incompatibility between needs and interventions; widened disparities  **[Outcomes]** risk perpetuating hierarchies of gender- and age-related power   - There is a problematic divergence between what adult stakeholders [A] expect children (particularly those from low-income areas) [A] to do with digital media [I] and with what they actually do [C], as well as between development interventions’ assumptions about the potential and the realities of many children’s [A]access to, skills with, and interest in ICT-related interventions [I]. Too often, interventions have tended to focus on the supply side while ignoring dynamics that structure demand and use [C] and, therefore, condition practices; they thereby risk perpetuating hierarchies of gender- and age-related power [O].   **[Disabling conditions]** Global fascination with ICTs; proprietary ICTs  **[Triggered Mechanisms]** *raising normative expectations* to be part of global markets that are impossible to meet in their rural location; *dependence* on the terms of service of the ICT provider and state regulations  **[Outcomes]** Lack of long-lasting social change; tools for state censorship and surveillance   - However, few if any cases show evidence of long-lasting social change [O]. Through the fascination with ICTs [C]: “the global corporate players (through new gadgets, schemes, and advertisement), as well as the government, through rhetoric and development schemes, are raising normative expectations to be part of global markets that are impossible to meet in their rural location with infrastructural limitations [M]. - ICTs are usually proprietary [C], leaving programme providers dependent [M] on the terms of service of the ICT provider (cost, data security, user privacy, commercial exploitation of data, etc.). Further, ICTs are regulated [M] by state bodies [A] either directly or through corporate franchises, and quite often in a restrictive manner involving censorship, and can be used as tools for surveillance [O].   **[Enabling conditions]** understanding of how technology interacts with social, political, and economic factors; evaluate ICTs alongside the non-digital development opportunities relevant to children and their life contexts.  **[Triggered Mechanisms]** Promote critical assessment, break assumptions  **[Outcomes]** Maximise potential of ICTs   - Maximising the positive potential [O] of ICTs [I]requires a grounded understanding of how technology interacts with social, political, and economic factors important for development [C]. - The role of ICTs needs to be critically assessed, rather than assumed [M], and evaluated alongside the non-digital development opportunities relevant to children and their life contexts [C]. |
| 24_  New forms of adolescent voice and agency through ICT and mobile phone use. | Mobile phone and internet use | School-going adolescents attending class VIII, who were mostly 14 years old  Dhaka, Bangladesh  Gatekeepers(teachers, and the government) | **[Enabling conditions]** Higher SES; owning devices; access to computers in schools; ICT used in school curriculum; unlimited internet access  **[Triggered Mechanisms]** opportunity for learning  **[Outcomes]** research, education, and skill building  SES / ownership of ICT devices   - Although everyone has access to a mobile phone, irrespective of class, location, and gender, the quality of access varies depending on whether the devices are personally owned by the adolescent [A] or shared with family members [A] - In addition to owning their devices [C], adolescents belonging to the higher socio-economic groups [C] can use computers in school [C], and much of their curriculum is taught [C] using ICT [I]. Unlimited access [C] to the internet [I] also allows them to spend more time on the internet for different purposes, such as research, education, and skill building—an opportunity that other adolescents [A] do not have [O].   **[Enabling conditions]** Access to information and greater exposure to various ways of being and doing.  **[Triggered Mechanisms]** Increased self-awareness, self-confidence.  **[Outcomes]** Increased voice and agency; hone interpersonal skills, and contribute to decision-making.  Voice and agency   - Our study shows that male and female adolescents [A] have gained voice [O] that they did not have before through mobile and internet use [I]. Armed with information and greater exposure to various ways of being and doing [C], they form and express their own discover hidden talents that they themselves were unaware of [O]. Finally, they are able to hone interpersonal skills, and contribute to decision-making on certain matters at the household level [O]. These processes lead processes lead to incremental increases in self-confidence [M] of the adolescents, which has a positive impact on adolescent voice and agency [O].     **[Disabling conditions]** Lower SES; limited access to ICT and devices  **[Triggered Mechanisms]** Reduced opportunity for learning; reduced autonomy/agency; choice-making between education and entertainment  **[Outcomes]** use of ICT may be limited to entertainment purposes   - That is why these adolescents (lower SES) [A] use it mostly media, downloading songs and movies, or for gaming; they use the internet for educational purposes only when necessary [O]. Therefore, although these adolescents know about the various ways the internet may be used for educational purposes, their actual use may be limited to entertainment purposes [O] because of the short time they get to use the device [C]. In Bengali medium, rural and slum schools, although ICT education is compulsory from class VI onwards, adolescents do not always have access to computers or have to share them. This reduces their opportunity to use them for educational purposes and develop adolescents who own devices have the freedom to visit any site they wish [M].   **[Disabling conditions]** Girls’ use and access to ICT heavily restricted; Biases and negative assumptions regarding digital devices and connectivity  **[Triggered Mechanisms]** Reduced autonomy/agency (girls>boys)  **[Outcomes]** gender differences in the use of the mobile phone  Gender   - There are gender differences in the use of the mobile phone [O] even when both girls and boys have access. Girls from middle-class and poorer socio-economic groups [A] and particularly girls from the rural area are heavily restricted [C] by parents [A] in their use of phones   Gatekeepers   - Biases and negative assumptions regarding digital devices and connectivity [C] among gatekeepers [A], both at home and in school [context], play a pivotal role in shaping the quality of access [M] and determining how adolescents use digital technology [O]. Most parents [A] have a negative view of adolescents using a mobile phone and internet. - Similar biases are seen among school teachers [A] as well. Rural school teachers believe that boys are more interested in, and better at, using computers. Consequently, they are given more opportunity to work directly on the computers. Adolescent girls, who are already disadvantaged in terms of digital access compared to the boys, are therefore facing additional challenges [O] because of the negative biases [C] of both parents and teachers [A]. |
| 25_  ICT, youth and urban governance in developing countries: Bangladesh perspective. | ICT and mobile platforms | Youth, college students  Bangladesh | **[Enabling conditions]** capacity of mobile platforms providing daily exchanges.  **[Triggered Mechanisms]** Connectivity  **[Outcomes]** pace and volume of exchange expanded; youth-local government relations brought closer.   - Another dimension of youth-local government relations [O] related to capacity concerns the volume of traffic that mobile platforms in particular have brought to the citizen-government relationship [C]. Contacts that were once annual or monthly are now daily in frequency and the pace and volume of exchange will expand [O] with the increased access to ICT devices [C].   **[Disabling conditions]** factors such as technological and human capacity, financial sustainability and bureaucratic resistance  **[Triggered Mechanisms]** disconnect; limit the adoption of ICT programs  **[Outcomes]** reduced their long-term impact of e-Gov services; youth-local government ICT gap + low confidence.  Widening youth-local government ICT gap   - The interviews provided clear evidence of disconnect [M] between young activists [A] found in most posts of confidence [O] in local government [A]. Experience with e-Government services in the past has demonstrated that factors such as technological and human capacity, financial sustainability and bureaucratic resistance [C] can limit the adoption [M] of ICT programmes [I] and reduce their long-term impact [O]. - The reality is that most people in government are not very strong ICT users [C]. A key challenge reported on by youth interviewees is the tendency of governments to limit their view of ICTs and youth to issues of access and skills [C]. Young people are interested in applying their skills to influence social, economic and political arenas [O].   **[Enabling conditions]** Mobile phones [[I] connected to social media; new channel of communication; fast-moving changes of ICT; pervasive presence of mobile platforms; inventiveness and adaptiveness of young people  **[Triggered Mechanisms]** space for expressing voice; dynamic boundaries of ICT are being pushed forward on a daily basis  **[Outcomes]** engage local government, contribute to this broader goal of developing, democratic institutions for youth; transformation of governance   - Mobile phones [[I] connected to social media [C] allow young people [A] to engage local government [O] by expressing voice [M]. - ICT is not the answer to developing democratic institutions for youth [O] in the developing world but represent an entire new channel of communication [C] that may contribute to this broader goal [O]. - governance itself is transformed [O] by fast-moving changes of ICT [C] in the hands of the young [A]. For one thing dynamic boundaries of ICT are being pushed forward on a daily basis [M] because of the pervasive presence of mobile platforms [C] and the inventiveness [C] of young people [A] in adapting [C] technological devices to meet their needs [O].   **[Enabling conditions]** limitations of broadband  **[Triggered Mechanisms]** Ease of engagement; innovation  **[Outcomes]** levelling influence in the rural–urban divide; low-end phone capabilities and simple Internet access facilitating scaled-down versions of social networking, pay-as-you go mobile data access and searching.   - Mobile platforms [I] have a levelling influence in the rural–urban divide [O]. Many applications [I] are making it easier to engage [M] in communication, education, health care and businesses from rural settings. The limitations of broadband [C] have led to the development of innovative [M] ‘narrowband’ mobile communications applications [I] tailored for users in developing countries [O]. Low-end phone capabilities such as text messaging and simple Internet access [I] is facilitating scaled-down versions of social networking, pay-as-you go mobile data access and searching [O] |

| 26_  Design Lessons from Creating a Mobile-based Community Media Platform in Rural India | IVR (Interactive Voice Response) systems  Mobile phones | Rural communities  India  [IVR operator]  Mobile Vaani  Local partner NGOs  In-house community mobilization team  Community volunteers  Volunteer clubs | **[Enabling conditions]** Freedom of expression  **[Triggered Mechanisms]** Agency and voice; empowerment  **[Outcomes]** Stimulate community discussions and push for government action; Increased individual knowledge   - Mobile Vaani [I] is like an ideal community media platform where people [A] represent themselves the way they want to [C]. […] with a view to encourage further discussion [O] within the community or make a dent in government administration [O]. - Impact at an individual level seems to happen either directly when information is posted on the platform about new opportunities that people can avail [O], or in a softer manner when users begin to feel empowered [M] because of the self-expression outlets provided by MV [C].   **[Enabling conditions]** Contextualized message with local references and local language.  **[Triggered Mechanisms]** Association and familiarity  **[Outcomes]** Relevant content created   - One of the key benefits of participatory content production [O] is that it helps contextualize the message well – the content contains local references, it is in the local language [C], and people are therefore able to associate with it more easily [M]   **[Enabling conditions]** Involving stakeholders, knowledge sharing platform; rendezvous point  **[Triggered Mechanisms]** *Networked connections*  **[Outcomes]** Direct reach to communities, social accountability   - MV [A] identified local partner NGOs [A] who actively reached out to communities in which they were working [O] - An in-house community mobilization team [A] was created with staff who recruited volunteers from among diverse user groups, and the volunteers in turn brought their communities on to the platform. - Volunteer network was built [O] into a hierarchical structure with the formation of volunteer clubs [A] at the district level. - When different stakeholders are a part of the same community [C], local media systems can help enforce [M] checks and balances [O] between them and build social accountability [O]. Media also plays other important roles such as to enable knowledge sharing [C] and to provide a rendezvous point for featuring cultural and traditional activities [C].   **Community-Institutional relationship**  **[Enabling conditions]** grievances made public  **[Triggered Mechanisms]** public exposure; leverage on positive relationship  **[Outcomes]** improve the success rate of making the authorities more accountable; maintain positive relationship between the volunteers and the administration; ensure resolution rather than antagonism.   - grievances are made public on the forum [C] instead of just being registered as an individual complaint on a hotline or a government department. This public exposure [M] seems to improve the success rate by making the authorities more accountable [O] - MV [I] further endeavors to maintain a positive relationship between the volunteers and the administration [O], and leverage this rapport [M] to ensure resolution rather than appear antagonistic [O].   **[Enabling conditions]** offline processes  **[Triggered Mechanisms]** organizational driver  **[Outcomes]** drive technology adoption, build credibility for the system in the eyes of the community, and provide a bi-directional communication conduit   - The relevance of offline processes to drive technology adoption, build credibility for the system in the eyes of the community, and provide a bi-directional communication conduit [O] between the organization and its users [A], emphasizes the need to institutionalize these processes.   Theory   - *The theory of change for development through Community Media (CM)can be categorized along four broad pathways. First, being able to train people to create content for their communities and initiate discussions, ensures that the content is contextually relevant for the community. Second, being able to ensure representativeness on CM for marginalized groups across caste, class and gender lines empowers them to voice themselves, which apart from making them more confident about themselves also enables them to challenge local power structures. Third, empowering people to talk about their problems and concerns on an open media platform helps promote good governance and accountability through checks and balances that civil society actors are able to impose on institutions. Fourth, CM also plays a role in community building by providing a forum for people to share their views and cultural expressions, thereby bringing communities closer through articulation of a shared identity.* |
| --- | --- | --- | --- |

**TEMPLATES:**

**[Enabling conditions]**

**[Triggered Mechanisms]**

**[Outcomes]**

**[Disabling conditions]**

**[Triggered Mechanisms]**

**[Outcomes]**

| POTENTIAL WORKING THEMES |
| --- |
| **Theme: Progressive sense of place as resource (AGENCY)**  **Theme: Coordination and female/youth Leadership (AGENCY)**  **Theme: Knowledge as Power (AGENCY)** |
| **Theme: Identity and representation (VOICE)**  **Theme: Involvement of primary stakeholders (VOICE)** |
| **Theme: Facilitative resources**  **Theme: Engaging/appropriate content**  **Theme: Privacy** |
| **Theme: ICT and its possible threats to youth SRHR** |

Models mentioned in literature


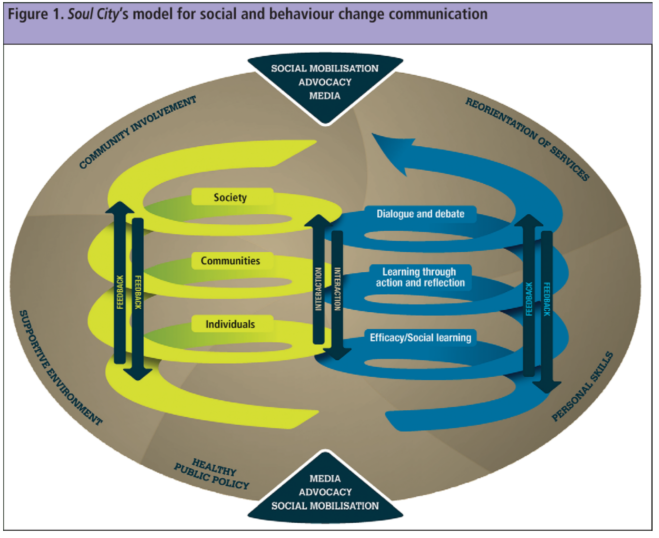

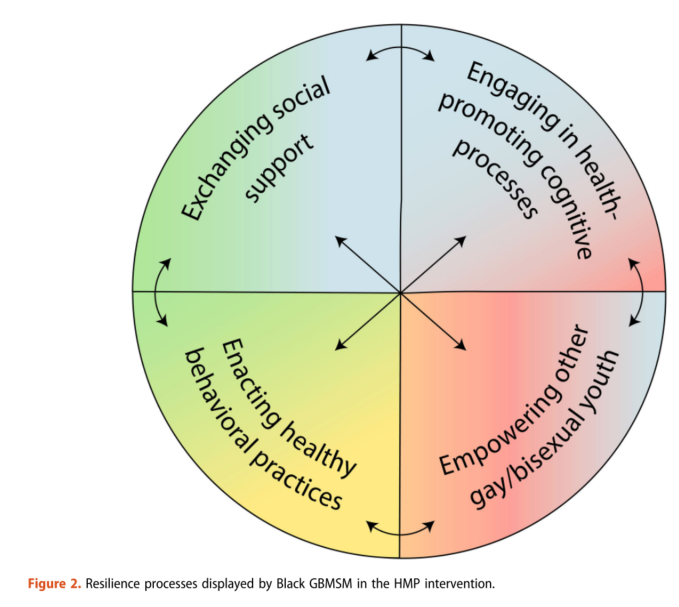

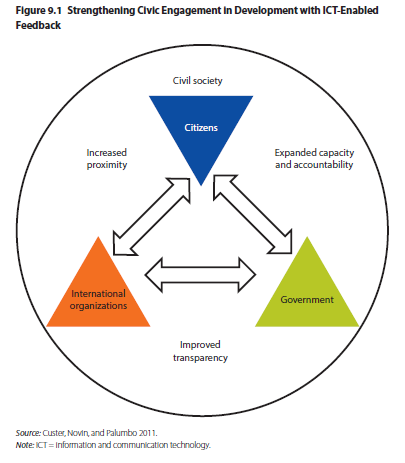

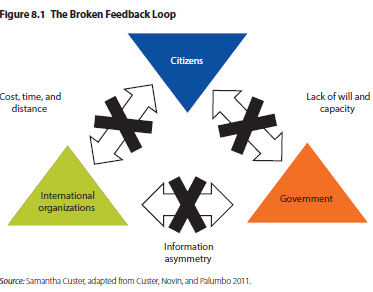


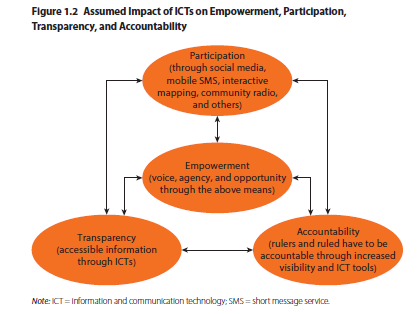

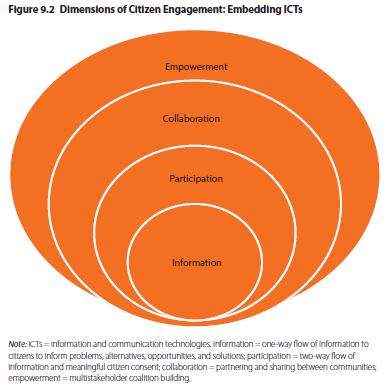

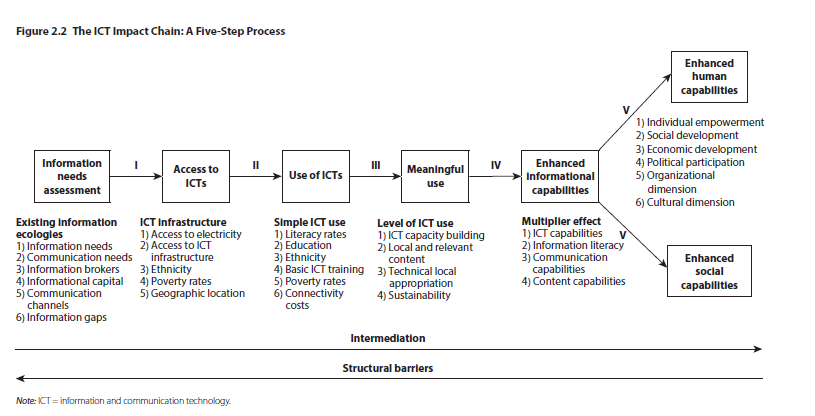


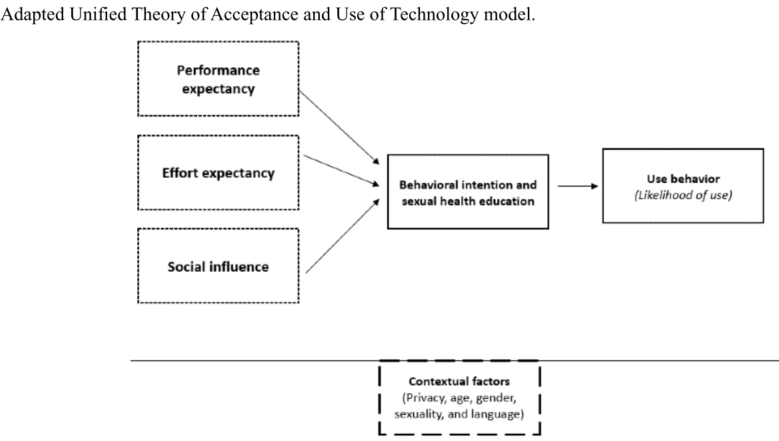

Supplement: Supplementary file 3 — Supplementary file3 (DOCX 794 KB) [file 11524_2022_678_MOESM3_ESM.docx]
